# Proteomics of a fuzzy organelle: interphase chromatin

**DOI:** 10.1002/embj.201387614

**Published:** 2014-02-17

**Authors:** Georg Kustatscher, Nadia Hégarat, Karen L H Wills, Cristina Furlan, Jimi-Carlo Bukowski-Wills, Helfrid Hochegger, Juri Rappsilber

**Affiliations:** 1 Wellcome Trust Centre for Cell Biology, University of EdinburghEdinburgh, UK; 2 Genome Damage and Stability Centre, University of SussexBrighton, UK; 3 Department of Biotechnology, Technische Universität BerlinBerlin, Germany

**Keywords:** Cdk regulation, chromatin, machine learning, organelle, proteomics

## Abstract

Chromatin proteins mediate replication, regulate expression, and ensure integrity of the genome. So far, a comprehensive inventory of interphase chromatin has not been determined. This is largely due to its heterogeneous and dynamic composition, which makes conclusive biochemical purification difficult, if not impossible. As a fuzzy organelle, it defies classical organellar proteomics and cannot be described by a single and ultimate list of protein components. Instead, we propose a new approach that provides a quantitative assessment of a protein's probability to function in chromatin. We integrate chromatin composition over a range of different biochemical and biological conditions. This resulted in interphase chromatin probabilities for 7635 human proteins, including 1840 previously uncharacterized proteins. We demonstrate the power of our large-scale data-driven annotation during the analysis of cyclin-dependent kinase (CDK) regulation in chromatin. Quantitative protein ontologies may provide a general alternative to list-based investigations of organelles and complement Gene Ontology.

## Introduction

Some of the first achievements of proteomics were to define the protein composition of organelles following isolation or quantitative enrichment (Mootha *et al*, 2003; Schirmer *et al*, 2003). A second generation of strategies has studied subcellular compartments based on co-fractionation with marker proteins on density gradients (Andersen *et al*, 2003; Dunkley *et al*, 2004; Foster *et al*, 2006). Defining organelles in this way critically depends on biochemical procedures and inherently introduces a series of purification artifacts. We have recently circumvented some of these by an approach called Multiclassifier Combinatorial Proteomics (MCCP; Ohta *et al*, 2010). A common feature of these investigations is that they attempt to completely separate genuine components from contaminants through biochemical and/or bioinformatics approaches. Crucially, the underlying assumption is that definite component lists can accurately describe complex biological structures. In light of the dynamic nature of organelles, an alternative concept may be needed. Through examination of human interphase chromatin, we develop an approach to capture the dynamic composition of biological structures, rather than enforcing static binary protein annotation.

Our analysis of interphase chromatin followed a three-stage process. (i) We developed a new protocol to biochemically isolate chromatin-enriched fractions. (ii) We employed MCCP (Ohta *et al*, 2010) with a refinement to encapsulate different degrees of functional involvement of proteins in chromatin. (iii) We then derived for each protein its probability of having a general chromatin-based function. The final result is a quantitative protein ontology term “interphase chromatin” that complements manually curated Gene Ontology (GO; Ashburner *et al*, 2000) and network-extracted ontology (NeXO; Dutkowski *et al*, 2013). We then apply this method to analyze changes in chromatin mediated by Cdk1 and Cdk2 cyclin-dependent kinase (CDK) activities in S-phase, and identify novel cell cycle-regulated chromatin proteins that play a role in S-phase entry and progression.

## Results and Discussion

### A new chromatin enrichment procedure

**Figure 1:**
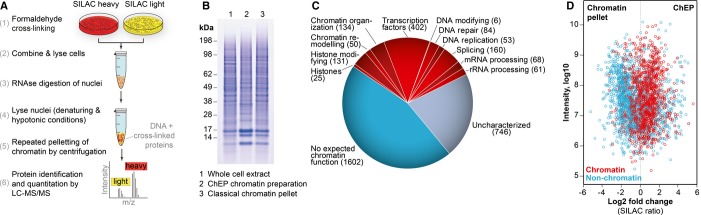
Chromatin enrichment for proteomics (ChEP).
Outline of the ChEP procedure, see Materials and Methods for details.SDS–PAGE gel of a typical chromatin fraction obtained using this procedure, compared to a whole-cell lysate and a classical chromatin pellet.Proteomic analysis of a typical ChEP chromatin sample. 3522 proteins were identified and classified manually according to their function. The number of proteins per category is indicated in brackets.SILAC-based quantitative proteomics comparing ChEP chromatin with a classical chromatin pellet, demonstrating that ChEP enriches for chromatin players more efficiently. The 1024 known chromatin proteins (red) and 1706 proteins with no expected chromatin function (blue) were annotated manually based on literature evidence. Uncharacterized proteins are not shown.

As a first step, we optimized the proteomic coverage of human interphase chromatin, that is, the DNA/histone fiber and all proteins associating with it. For this, we developed a new procedure, which we call chromatin enrichment for proteomics (ChEP). We fix proteins in chromatin by *in vivo* formaldehyde cross-linking and remove non-covalently associated proteins by washing under extremely stringent conditions (Fig 1 and Materials and Methods). These initial conditions relate to standard chromatin immunoprecipitation (ChIP) experiments (Solomon *et al*, 1988) and were also employed as starting point of a recent proteomic analysis of telomeres (Déjardin & Kingston, 2009). However, our approach then uses simple centrifugation to collect whole chromatin for subsequent mass spectrometric analysis of the associated cross-linked proteins. This should allow quantitative analyses of processes that affect chromatin globally.

In a representative proteomic analysis, we identified 3522 proteins comprising typical chromatin-associated processes such as transcription, histone modification, and DNA repair (Fig 1C). The protocol enriches for chromatin factors in a considerably more efficient way than the classical chromatin pellet (Fig 1D). Nonetheless, nearly half of the proteins identified at this proteomic scale have no apparent chromatin-related function. Additional DNA-directed isolation steps, such as hydroxyapatite chromatography, failed to reduce co-purifying unrelated proteins further (not shown).

For mitotic chromosomes, we achieved a seemingly clear-cut separation of genuine components and purification background using MCCP (Ohta *et al*, 2010). However, such a separation may be artificial in the case of interphase chromatin. A multitude of highly dynamic and regulated biological processes take place in interphase chromatin, such as replication, gene expression, and DNA repair. Many proteins only associate with chromatin under specific physiological conditions. For others, only part of their cellular pool is active in chromatin. To emphasize these dynamic aspects of interphase chromatin, we call it a “fuzzy” organelle. The ability to describe these varying degrees of contribution is essential for an accurate understanding of chromatin plasticity. Rather than qualitatively separating chromatin from background proteins in ChEP fractions, we therefore aimed to quantify the contribution of each protein toward chromatin. We hypothesized that MCCP would also be able to address this.

### A novel type of classifier to infer protein function

**Figure 2:**
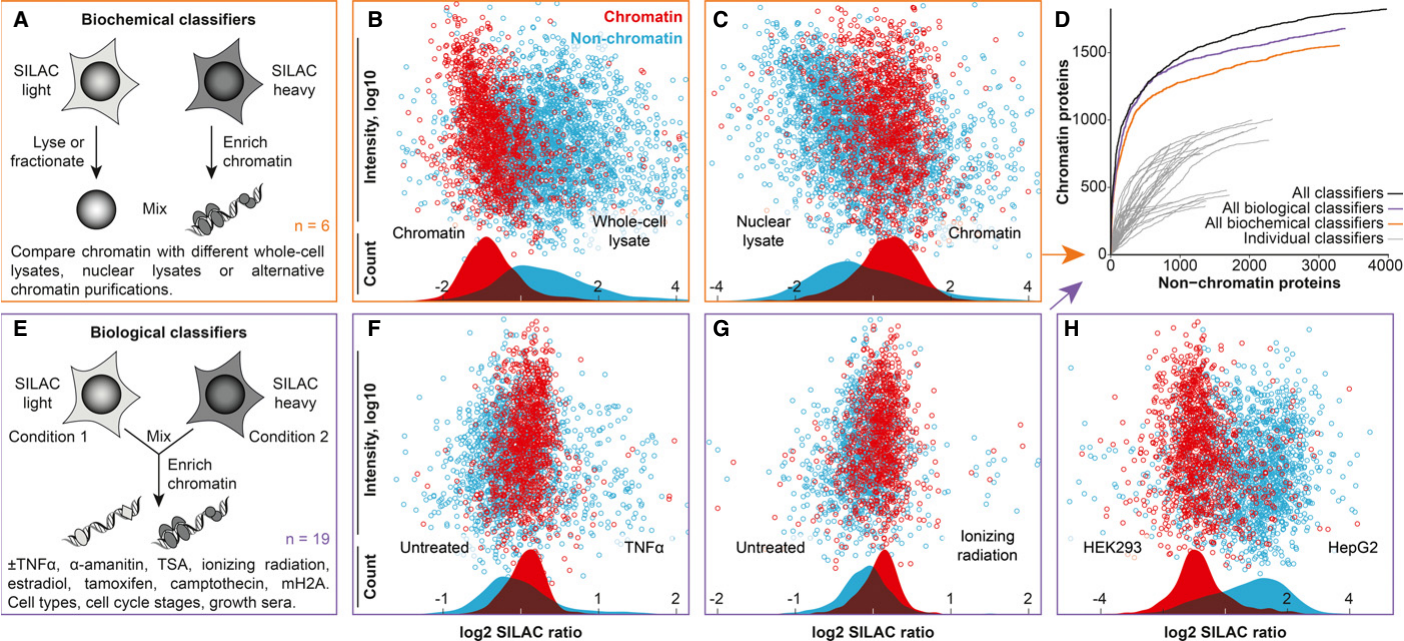
Defining interphase chromatin through biochemical or biological classifiers. A Traditional biochemical classifiers compare chromatin with other subcellular fractions. B, C SILAC plots showing enrichment of known chromatin (red) over non-chromatin proteins (blue) for two such experiments. The distributions of chromatin and non-chromatin proteins are displayed as overlaid graphs. D Individual experiments are integrated by machine learning for a more powerful distinction of chromatin and non-chromatin proteins, as assessed by receiver operating characteristic-like curves. E–H Schematics and SILAC plots of representative biological classifiers showing the uneven distribution of chromatin and non-chromatin proteins in chromatin fractions prepared from distinct physiological conditions (see Table 1 for full list of experiments). The following number of proteins were quantified and plotted after median normalization: (B), 1441 chromatin proteins/2882 non-chromatin proteins; (C), 1373/2636; (F), 1130/1709; (G), 933/1193; (H), 1156/1774.

MCCP combines multiple “classifier” experiments, which individually separate two protein groups incompletely, into a powerful super-ranking using a random forest (RF; Breiman, 2001) machine learning algorithm. This algorithm learns to distinguish between chromatin-like or chromatin-unlike behavior in classifier experiments on the basis of well-described training proteins. Classifier experiments used here include standard approaches such as comparing a chromatin-enriched fraction with whole-cell lysates or other subcellular fractions (Fig 2A–C and Table 1). We applied six “biochemical classifiers” that infer function from physical association and invariably carry the risk of purification artifacts (contaminants and losses). Moreover, these classifiers do not take into account different physiological states of chromatin and consequently cannot integrate regulated changes in chromatin composition. To circumvent this problem, we developed an alternative type of classifier experiment. We compared ChEP preparations differing as a result of regulated physiological changes within the cell. This “biological classifier” approach aims to reduce purification artifacts while at the same time encapsulating biological complexity (Fig 2E). Function is inferred by machine learning from co-behavior with known reference proteins. In our case, these are 486 proteins linked to chromatin and 582 not (see Materials and Methods).

**Table 1:** Experiments used to infer the composition of interphase chromatin

ID	Sample 1 (SILAC light)	Sample 2 (SILAC heavy)	Sample 3 (SILAC medium)	Cell line	Proteins	Comment
Biochemical classifier experiments (comparing ChEP chromatin with other biochemical fractions)						
a	Whole-cell lysate	ChEP chromatin	/	MCF-7	5227	See note 1
b	ChEP chromatin	Whole-cell lysate	/	HeLa	5650	See note 1
c	Whole-cell lysate	ChEP chromatin	/	HepG2	2262	
d	Nuclei lysate	ChEP chromatin	/	HeLa	5121	
e	Chromatin pellet	ChEP chromatin	/	HeLa	4852	
f	Chromatin pellet	ChEP chromatin	/	HepG2	1676	
g	Whole-cell lysate	ChEP chromatin	/	HeLa	5231	Label-swap of (b)
Biological classifier experiments (comparing ChEP fractions from different biological conditions)						
a	Untreated	TNF-α, 5 min	/	HeLa	3789	
b	Untreated	TNF-α, 10 min	/	HeLa	3658	
c	Untreated	TNF-α, 30 min	/	HepG2	3615	
d	α-Amanitin	DMSO	Trichostatin A	MCF-7	1546	
e	Untreated	Camptothecin	/	U20S	2769	
f	Untreated	Ionizing radiation, 10 Gy	/	U20S	2793	
g	Untreated	Ionizing radiation, 30 Gy	/	U20S	2742	
h	Untreated	E2 and 4-OHT	E2	MCF-7	2768	See note 2
i	From HEK293 cells	From HepG2 cells	/	/	3719	
J	Cell cycle Gl/S	Cell cycle phase M	Cell cycle phase G2	HeLa	3720	See note 3
k	10% rat serum 1	10% dialyzed FCS	/	HepG2	3038	See note 4
l	10% rat serum 2	10% dialyzed FCS	/	HepG2	3297	See note 4
m	Untreated	1 μg/ml doxycycline	/	HeLa	2271	See note 5
n	Untreated	1 μg/ml doxycycline	0.1 μg/ml doxycycline	HeLa	1843	See note 5
o	α-Amanitin	DMSO	Trichostatin A	MCF-7	1517	Replica of (d)
p	Cell cycle phase G2	Cell cycle Gl/S	Cell cycle phase M	HeLa	4088	See note 6
q	Untreated	E2 and 4-OHT	E2	MCF-7	3394	Replica of (h)
r	E2 and 4-OHT	E2	Untreated	MCF-7	2883	Label-swap of (h)
s	E2	Untreated	E2 and 4-OHT	MCF-7	1301	Label-swap of (h)
t	E2 and 4-OHT	E2	Untreated	MCF-7	3481	Label-swap of (h)
u	E2	Untreated	E2 and 4-OHT	MCF-7	2914	Label-swap of (h)
v	Untreated	1 μg/ml doxycycline	/	HeLa	3123	Replica of (m)
w	1 μg/ml doxycycline	Untreated	/	HeLa	3187	Label-swap of (m)
Additional classifier experiments						
a	Purified w/o RNase	Purified with RNase	/	HepG2	1749	See note 7
b	Cell cycle Gl/S	Cell cycle phase M	/	HeLa	1183	5 min fixation
c	Cell cycle Gl/S	Cell cycle phase M	/	HeLa	1196	10 min fixation
d	Cell cycle Gl/S	Cell cycle phase M	/	HeLa	1273	15 min fixation
e	Cell cycle Gl/S	Cell cycle phase M	/	HeLa	1372	20 min fixation

Note 1 Mixed 1:4 to increase detection of chromatin factors (affects all proteins, so no impact on classifier performance).

Note 2 E2 is 17-β-estradiol; 4-OHT is 4-hydroxytamoxifen.

Note 3 Cells arrested by thymidine (Gl/S), RO-3306 (G2), nocodazole (M).

Note 4 Rat serum replaced FCS in cell culture medium.

Note 5 Stable cell line expressing macroH2Al.l from a doxycycline-induced Tet-ON promoter.

Note 6 Label-swap replica of (j), but only fixed for 5 min with formaldehyde.

Note 7 Standard ChEP procedure includes RNase treatment, see Materials and Methods.

Our biological classifiers rely on global, systemic perturbations. This ensures that a broad range of chromatin processes is affected and in many ways, even though such changes might be subtle. For example, ChEP preparations from cells treated with or without TNF-α show subtle, global changes that affected chromatin and non-chromatin proteins differently (median 1.12-fold difference; Fig 2F). We subsequently added data on chromatin-enriched fractions from another 18 different biological conditions, including cell types, cell cycle phases, and drug treatments (see Table 1 for full list). In all these cases, we observe general, coordinated alterations, such that the densities of chromatin and non-chromatin proteins vary slightly throughout all SILAC plots (Fig 2F–H). Even though detectable through the high accuracy of quantitative proteomics (Ong *et al*, 2002), such small bulk changes are usually overlooked or dismissed in favor of large changes in few proteins. For some experiments, no coordinated differences between chromatin and non-chromatin proteins were observed, for example protein turnover (not shown), and these were not included here.

We integrate co-fractionation changes in response to many *in vivo* perturbations instead of suggesting function from biochemical co-fractionation alone. As a consequence, the composition of the organelle is defined in its native environment. Accordingly, abundant contaminants of chromatin purifications are correctly identified as false positives by biological classifiers, since these proteins do not respond to physiological changes in the same way as genuine chromatin components (Supplementary Fig S1). Note that a virtually unlimited number of biological classifiers can be conceived. Even treating cells with TNF-α for 5 min rather than 10 min provides additional information (Supplementary Fig S2). Importantly, perturbations do not need to target the structure in question directly or selectively, as long as they induce global biological changes that affect the structure.

### An integrated chromatin score

The output, an integrated chromatin score, was validated using 5795 proteins that we manually annotated as either “chromatin proteins” (any reported function on chromatin) or “non-chromatin proteins” (well-characterized proteins without indication of involvement with chromatin; Fig 2D). Notably, the combined set of global perturbation experiments discriminates chromatin from non-chromatin players better than a classic biochemical enrichment experiment, such as comparing a chromatin fraction with a whole-cell lysate (Supplementary Fig S1). For the remainder of this study, we integrated all experiments that showed some bulk separation (see Table 1). This optimized performance as judged by receiver operating characteristic (ROC)-like curves (Fig 2D) and maximized the number of proteins observed.

### From machine learning score to interphase chromatin probability

**Figure 3:**
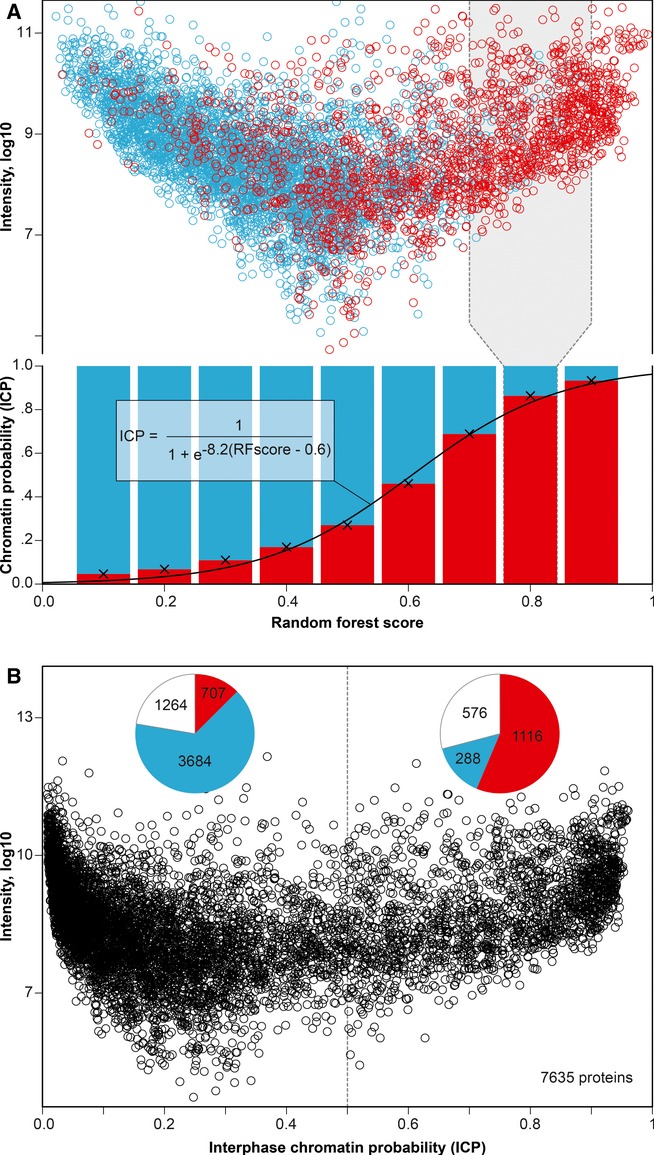
Toward a probabilistic chromatin definition.
Using 5795 proteins of known function, the percentage of chromatin (red) and non-chromatin proteins (blue) was calculated for overlapping windows (e.g., gray box) of the random forest (RF) machine learning score. Fitting a sigmoid curve through the percentages translates the RF score into “interphase chromatin probabilities” (ICPs). 1823 evaluation proteins are known chromatin players, while 3972 have no expected chromatin function.Scatterplot showing ICPs for 7635 human proteins (the 5795 evaluation proteins and 1840 uncharacterized proteins). Pie charts show protein categories above and below ICP 0.5, including uncharacterized proteins (white).

A protein with integrated chromatin score of 0.8 received a chromatin vote from 80% of the trees in the RF. The score provides a ranking but gives no indication on how likely the protein has a chromatin function. To provide dimension and scale, we calibrated the score distribution making use of the 5795 annotated evaluation proteins in our dataset. We calculated the fraction of proteins with reported chromatin functions among all characterized proteins within score windows. We described the result as a sigmoid function (Fig 3A, see Materials and Methods for details). In this way, we integrate knowledge on proteins with similar scores into the probability of any given protein to have a chromatin function. This translation is robust and reproducible (Supplementary Fig S3). A calibrated score of 0.8 for instance means that eight of 10 reference proteins with this value have a reported chromatin function, thus providing a probability for the function of this protein. We refer to this value as interphase chromatin probability (ICP; Fig 3B, Supplementary Table 1). ICPs provide a general annotation on how similar a protein behaves experimentally to archetypal chromatin proteins. We provide ICPs for 7635 human proteins and protein isoforms, including the 5795 evaluation proteins (1823 proteins with literature evidence linking them to chromatin and 3972 non-chromatin proteins) and 1840 previously uncharacterized proteins. Proteins were classified as “uncharacterized” based on absence of literature but also had low GO coverage and weak domain-based prediction (Supplementary Fig S4). Of the 1840 uncharacterized proteins described in this study, 576 have a chromatin probability >0.5, indicating that hundreds of chromatin components are presently still uncharacterized. The large number of novel chromatin proteins is in line with a recent report that used alternative technology to test more than 100 proteins and found 42 previously unknown chromatin components (van Bemmel *et al*, 2013). ICPs integrate large-scale data for quantitative gene function prediction and can help systematically fill current annotation gaps.

### ICPs are consistent with the function of protein domains

To validate ICPs, we performed several tests based on literature knowledge and bioinformatics and finally applied the method to elucidate cell cycle regulation of chromatin. As a first validation step, we investigated the correlation between ICPs and the presence of protein domains that have been linked to interphase chromatin (Supplementary Fig S5). As expected, proteins with canonical chromatin domains (e.g., chromo, bromo, JmjC) invariably have high ICPs. Conditional and regulated chromatin proteins such as transcription factors with sequence-specific DNA-binding domains indeed cover a broad range of ICPs. Proteins with a Ras domain have no reported chromatin involvement and receive low ICPs. This suggests that ICPs can capture a dynamic range of involvement in chromatin.

### ICPs capture diverse biological behavior of proteins

**Figure 4:**
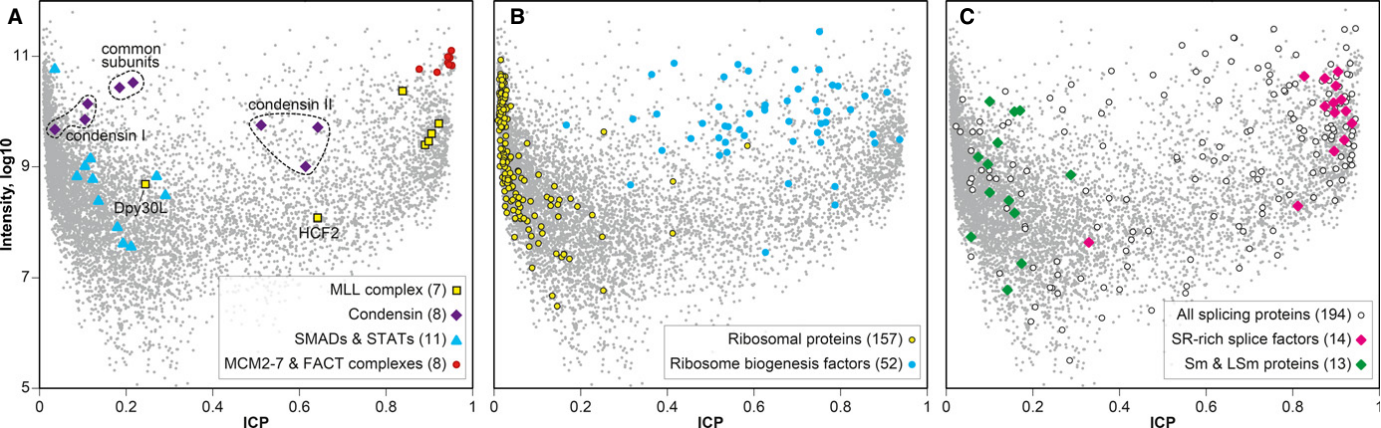
Interphase chromatin probabilities describe chromatin plasticity. A Protein complexes can have uniform interphase chromatin probabilities (ICPs; MCM2–7, FACT) but also heterogeneous ICPs (MLL, condensin) in agreement with additional roles of individual subunits. Consistently low ICPs are assigned to cytoplasmic, signal-dependent SMAD/STAT transcription factors. B, C ICPs also set apart ribosome biogenesis, which happens in nucleoli as part of or vicinal to chromatin, from core ribosome components (B), as well as core splicing factors (Sm/LSm) from SR proteins, which can act co-transcriptionally (C). Data information: The number of proteins in each group is indicated in brackets.

We next investigated whether ICPs accurately reflect the biological behavior of well-described proteins and their complexes. Typical chromatin-associated protein complexes such as MCM2-7 and FACT have consistently high ICPs for all their subunits (Fig 4A). In contrast, subunits with multiple functions correctly have different ICP values from core subunits. This includes the MLL histone methyltransferase subunits Dpy-30L (Xu *et al*, 2009) and HCF2 (Johnson *et al*, 1999; Fig 4A) and multiple NuRD components (Supplementary Fig S6). Different isoforms of NuRD subunits with redundant function receive similar ICPs, indicating large accuracy of ICP values. Ribosomes, commonly found contaminants in biochemical purifications, have low ICP values (Fig 4B). This indicates that ICPs successfully integrate biological rather than biochemical behavior. ICPs also match the dynamic chromatin association of the condensin complex (Fig 4A). In interphase, condensin I subunits diffuse into the cytoplasm (very low ICP) and condensin II subunits remain nuclear with a low chromatin affinity (Gerlich *et al*, 2006; medium ICP). Common condensin subunits show an intermediate behavior. This is consistent with ICPs being a parameter that describes the average behavior when multiple pools are present. Similarly, Cdk1 has a low ICP (0.15) as its main pool is bound to cytoplasmic cyclin B, while a minor fraction competes with Cdk2 for nuclear cyclin A and E and acts on chromatin (Santamaría *et al*, 2007). Different from ribosomes, factors of ribosome biogenesis associate with pre-rRNA co-transcriptionally and thus have some chromatin association. This is reflected in intermediate to high ICP values (Fig 4B). Interestingly, splicing factors show a large spread from low to high ICP values. This distribution is not random; all Sm and LSm proteins have low ICP values, while SR-rich splicing factors, which can act co-transcriptionally (Zhong *et al*, 2009), consistently have high ICP values (Fig 4C). Similarly, ICPs allow distinguishing canonical from conditional chromatin proteins. For example, SMAD and STAT transcription factors are normally absent from chromatin due to their signal-dependent nuclear localization and have consistently low ICPs (Fig 4A). In conclusion, ICPs provide a quantitative annotation that captures the subtle biological behavior of diverse proteins and functions, rather than providing classical “all or nothing” scores to distinguish between true and false positives.

### ICPs as quantitative annotation of the multifunctional proteome

**Figure 5:**
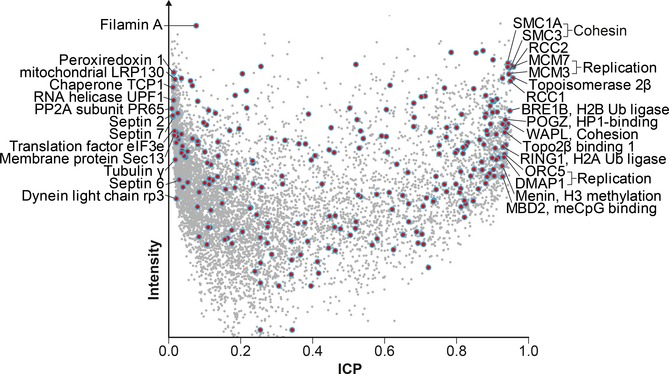
Interphase chromatin probabilities (ICPs) as quantitative annotation for multifunctional proteins. ICP distribution of 248 proteins with both cytoplasmic and chromosomal localization according to Gene Ontology (red/blue circles) reveals a protein's core function.

As a final test, we asked whether ICPs could identify predominantly chromatin-based proteins among those 248 proteins in our dataset that are both cytoplasmic and chromosomal according to the GO database (Ashburner *et al*, 2000). ICPs can successfully reveal these proteins' main activities as shown by the examples in Fig 5. Looking at the most extreme ICP values, 16 proteins with highest ICP are well-described chromatin proteins, while 13 proteins with lowest ICP have a main function elsewhere. Note that proteins with low chromatin ICP are not indicating GO artifacts, for example septin filaments interact with kinetochores during mitosis (Spiliotis *et al*, 2005; Zhu *et al*, 2008). This demonstrates that ICPs may help to address one of the large problems of protein annotation. Protein annotation databases are challenged by an increasing amount of data on proteins leading to an accumulation of proteins with multiple, apparently unrelated, annotations. For example, according to GO, 40% of all human nuclear proteins are also found in the cytoplasm. Many of these proteins will be multifunctional. However, increasingly sensitive analyses will decrease the value of protein localization for function prediction, for example chromatin proteins may be observed while they are translated in the cytoplasm. This ultimately reduces the value of such annotations. Quantitative protein ontologies, as suggested here, have the potential to solve these issues by providing a probabilistic dimension to protein annotations.

### ICPs as adjustable focus for Cdk-dependent chromatin regulation

**Figure 6:**
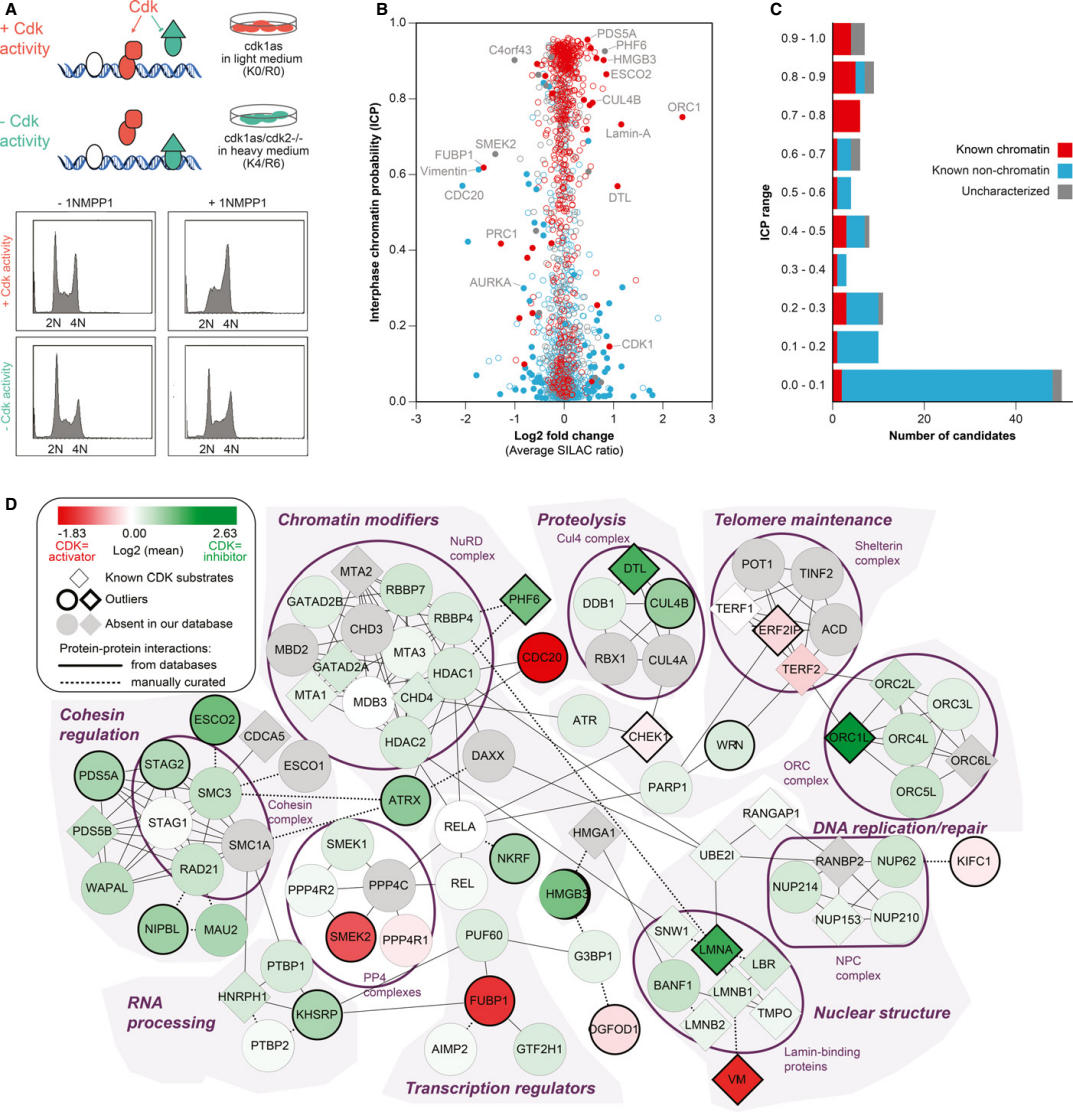
Using interphase chromatin probabilities (ICPs) to analyze Cdk-dependent changes in S-phase chromatin.
10^8^ cdk1as and cdk1as/cdk2^−/−^ DT40 cells, cultured in SILAC media, were incubated in 10 μM of 1NMPP1 for 2 h. Propidium iodide staining and flow cytometry analysis show the cell cycle profiles in the absence or presence of 1NMPP1. Cells were then collected and chromatin was extracted using ChEP method.The average SILAC ratios of 1652 proteins quantified in at least three Cdk experiments are plotted against their ICP. Filled circles represent significant Cdk outliers (*P *<* *0.05). Known chromatin proteins are red, known non-chromatin factors blue, and uncharacterized proteins gray.Bar chart showing how many of 114 conserved Cdk outliers fall in various ICP ranges. Color code as in (B).Protein network of candidates with an ICP > 0.5, all of which constitute core chromatin pathways. Candidates with increased chromatin binding after Cdk inhibition are shown in green, and candidates with decreased affinity in red. Gray signifies proteins that are in a known complex, but absent from our data. Bold borders mark significant outliers. Proteins with published Cdk phosphorylation sites are shown as diamonds.

ICPs could be used for guidance when looking for *bona fide* chromatin proteins. ICPs do not define specific chromatin functions of individual proteins. Therefore, we envision ICPs as a form of large-scale data-derived and quantitative GO term to allow focusing other datasets onto chromatin function. We undertook two studies to exemplify this. First, we analyzed changes in chromatin composition driven by Cdk-dependent cell cycle progression through S-phase (Fig 6A). Initiation and completion of DNA replication has a major impact on chromatin (Khoudoli *et al*, 2008), but how core chromatin processes are cell cycle regulated in somatic cells remains poorly understood. To address this question, we conducted a quantitative proteomics study that took advantage of an analogue-sensitive mutation in Cdk1 that we previously established in wild-type (WT) and Cdk2-knockout chicken DT40 cells (Hochegger *et al*, 2007). This mutation allows the rapid and highly specific inactivation of Cdk1 by the bulky ATP analogue 1NMPP1. Neither Cdk1 nor Cdk2 is required for S-phase progression, while inactivation of Cdk1 in Cdk2-knockout cells causes a complete block of DNA replication initiation and S-phase progression. Accordingly, after 2 hours of 1NMPP1 treatment, we observed an arrest in G1-and S-phase of the cell cycle only in Cdk2-knockout cells, while Cdk2-expressing WT cells proceeded to G2-phase (Fig 6A). Below, we will indicate the combination of Cdk1 and Cdk2 as “Cdk.”

**Figure 7:**
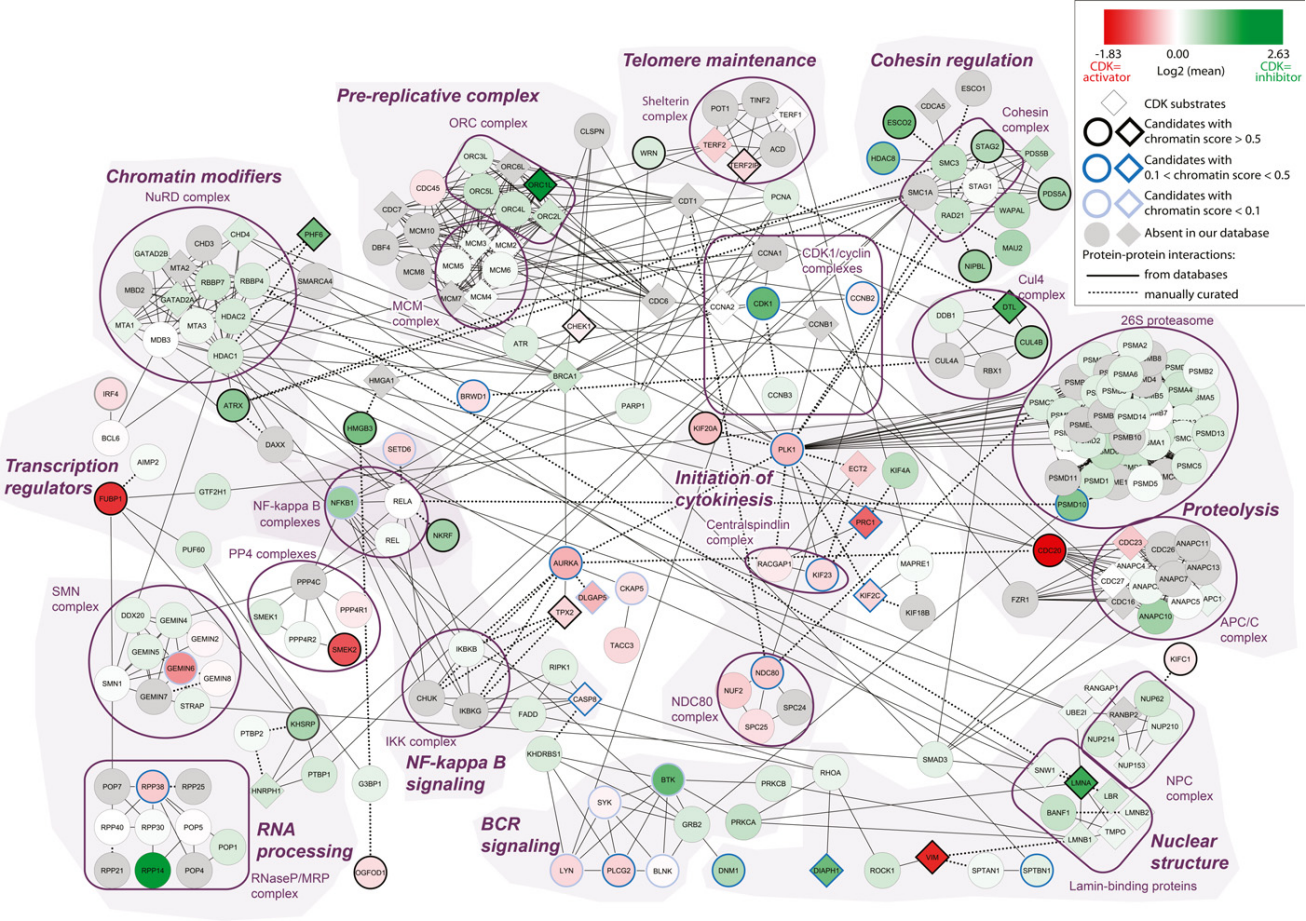
Protein network of candidates with an interphase chromatin probabilities (ICP) > 0.1. Candidates with increased chromatin binding after Cdk inhibition are shown in green; candidates with decreased affinity in red. Gray signifies proteins that are in a known complex, but absent from our data. Bold borders mark significant outliers, and colors indicate the range of ICP values. Known Cdk substrates are extracted from the Web site PhosphositePlus (http://www.phosphosite.org) and are shown as diamonds. To build the network, some pathways required the addition of outliers with an ICP below 0.1. All protein interactions are referenced in Supplementary Table 5.

We compared ChEP chromatin obtained from cdk1as and cdk1as/cdk2^−/−^ cells 2 h after Cdk1 inactivation. Statistical analysis of four independent experiments (including 2 label swaps) identified 135 proteins that showed a significant change in SILAC ratio among a total of 2402 proteins quantified in at least three experiments (Supplementary Fig S7 and Supplementary Table 2). Of these candidates, 114 had a human one-to-one ortholog (Supplementary Tables 2 and 3). As expected, Orc1, but not other Orc subunits, was strongly regulated by Cdk (Méndez *et al*, 2002) and so was the cohesin complex. However, a large number of cytoplasmic proteins were also affected by Cdk activity. This hampered the selection of novel candidates that participate in Cdk-regulated core chromatin processes based on SILAC ratios alone. We therefore took advantage of ICPs to filter our dataset of Cdk-regulated proteins according to their predicted functional association with chromatin (Fig 6B and Supplementary Table 3). We performed an in-depth protein network analysis using two different chromatin probabilities (Figs 6D and 7). A broad analysis was performed with proteins of ICP at least 0.1, and a more stringent filter was applied by using an ICP of 0.5. The low stringency ICP 0.1 filter removed already 50 of the 114 candidate proteins, most of which have no expected chromatin function (Fig 6C). This provided a clearer view of a complex dataset that includes loosely chromatin-associated processes such as NFκB and lymphocyte BCR signaling, SMN complex, cytokinesis and kinetochore components, as well as Cdk1 itself (Fig 7). The stringent filter provided a network that closely focuses on core chromatin processes such as chromatin modification, telomere maintenance, and nuclear structural organization (Fig 6D). Both networks provide useful insights into the cell cycle regulation of chromatin and reveal a variety of novel proteins that appear to be regulated in a Cdk-dependent manner.

### ICPs pinpoint novel Cdk-regulated chromatin players

**Figure 8:**
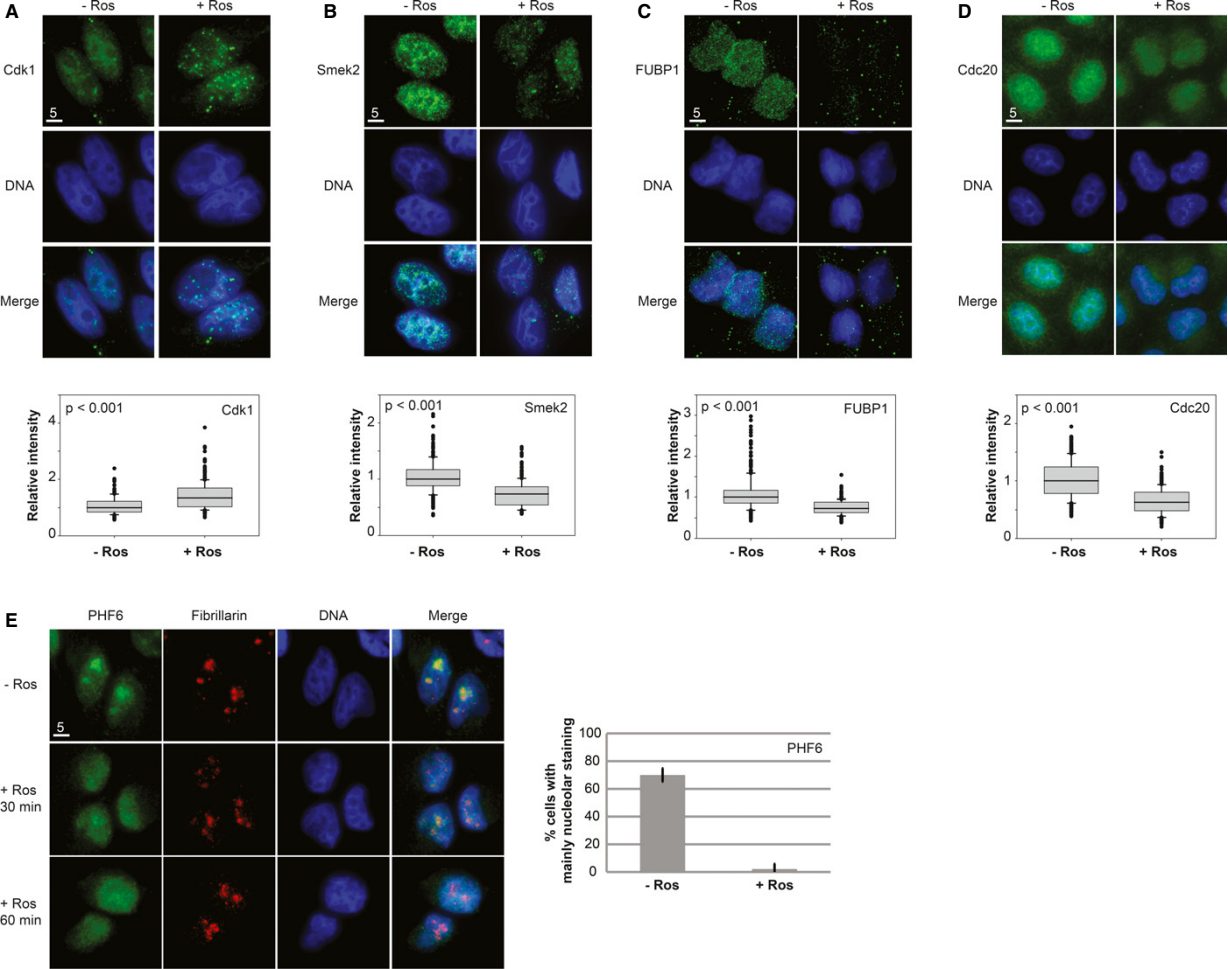
Validating Cdk-dependent chromatin association changes of selected outliers. A–E Validation of changes in chromatin affinity following Cdk inhibition of indicated candidates (2 h, 50 μM roscovitine) using Triton extraction and immunofluorescence (IF) in HeLa cells. Quantification of IF data was performed by measuring changes in signal intensities in Image J (A–D) and qualitative analysis of nucleolar staining (E). In each experiment, *n* > 50 nuclei were analyzed for each condition in three independent experiments (*P* indicates *P* value from Mann–Whitney test).

To demonstrate that we have indeed identified novel cell cycle-regulated chromatin factors, we experimentally validated five novel candidates for cell cycle-regulated chromatin association. We chose these candidates based on their combination of suggestive ICPs and high SILAC ratios. One is a known chromatin protein, the myc transcription regulator FUBP1 [ICP 0.62; reviewed in (Zhang & Chen, 2013)]. The PHF family protein PHF6 (ICP 0.93) is an uncharacterized protein implicated in Börjeson–Forssman–Lehmann syndrome (Lower *et al*, 2002). Smek2 (ICP 0.65) is a regulatory subunit of PP4 with cytoplasmic and nuclear localization (Chowdhury *et al*, 2008). Cdc20 is a regulatory subunit of the anaphase-promoting complex (Yu, 2007) that has so far not been implicated to function on interphase chromatin, but has a surprisingly high ICP (ICP 0.57) and shows a strong Cdk-dependent change in the SILAC experiments. We also included Cdk1 that has a low chromatin probability of 0.15, but that still is likely to have some chromatin-associated functions (Santamaría *et al*, 2007). We confirmed Cdk-dependent chromatin association of these proteins in human cells, before and after treatment with the Cdk inhibitor roscovitine, by Triton extraction and immunofluorescence. In each case, the candidate protein behaved as predicted by the SILAC data, either enhancing or decreasing their chromatin affinity after Cdk inhibition (Fig 8). In the case of PHF6, we observed a sequestration of the protein in the nucleolus, and a release onto the non-nucleolar chromatin after Cdk inhibition. In all other cases, we observed a simple increase or decrease in chromatin association following roscovitine treatment that corresponded with the respective SILAC ratio in the proteomic dataset.

**Figure 9:**
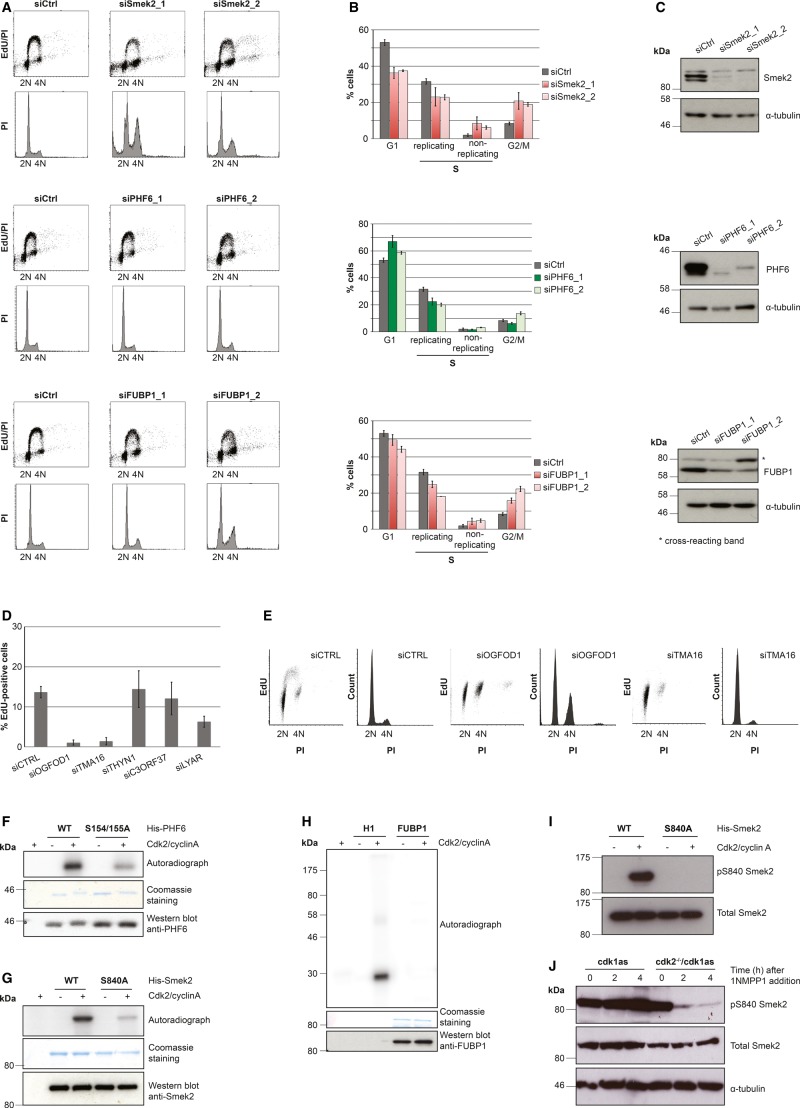
Cell cycle changes following depletion of candidate proteins and Cdk-dependent phosphorylation of Smek2 and PHF6. A Propidium iodide (PI)/EdU staining and FACS analysis of HeLa cell lines after 72 hours of depletion with indicated siRNAs. B Quantification of FACS cell cycle analysis in (A) showing the mean value and standard deviation of three independent experiments. C Verification of siRNA depletion of PHF6, FUBP1, and Smek2 by immunoblotting. D Quantification of the EdU incorporating fraction of RPE cells following 72 hours of depletion with siRNA pools against the uncharacterized proteins with high interphase chromatin probability (ICP) values (THYN1, ICP = 0.91; TMA16, ICP = 0.9; OGFOD1, ICP = 0.86; C3ORF37, ICP = 0.83; LYAR, ICP = 0.61). E FACS/PI histograms and EdU incorporation dot blots of cells 72 h after transfection with control, TMA16, and OGFOD1 siRNAs. F–H *In vitro* kinase assays with purified PHF6, Smek2, and FUBP1 and the indicated Smek2 and PHF6 mutant proteins. I Immunoblotting of recombinant WT and S840A Smek2 before and after incubation with ATP and Cdk2/cyclinA. J Immunoblotting of extracts of cdk1as and cdk2^−/−^/cdk1as DT40 cells at indicated time points following 1NMPP1 addition.

The observed Cdk-regulated chromatin association of these proteins could point to a role in cell cycle progression. We tested this hypothesis by analyzing the cell cycle profile following siRNA-mediated depletion of Smek2, PHF6 and FUBP1 (Fig 9A). In all three cases, we observed significant changes in the cell cycle profile after 72 hours of depletion of the candidates (Fig 9A). PHF6 knockdown caused an increase in the G1 population, while Smek2 and FUBP1 depletion caused a significant change in the replicative proportion of cells, suggesting functions for these proteins in S-phase progression (Fig 9B). Of these, the PP4 regulatory subunit Smek2 had the most dramatic phenotypes, suggesting important novel roles in cell cycle control. We then took advantage of the ICP resource to analyze a further set of five proteins with no known chromatin-associated function that nevertheless showed ICP values > 0.5 (Fig 9D,E) and a Cdk-dependent change in the SILAC ChEP experiments. The combination of high ICP and SILAC ratio could suggest that some of these proteins play a role in interphase progression. Indeed, we found significant cell cycle phenotypes for two of these five genes after knockdown in RPE cells. Both TMA16 and OGFOD1, both of which are largely uncharacterized proteins with no known function in cell cycle progression and chromatin biology (Saito *et al*, 2010; Wehner *et al*, 2010), show a strong reduction in cells in S-phase after 72 hours of depletion, suggesting the activation of cell cycle checkpoints in G1 and/or G2 or a direct block in replication initiation.

Our approach to observe changes in chromatin association following Cdk inhibition does not allow us to distinguish between direct Cdk-regulated targets and proteins that are indirectly affected by changes in cell cycle progression. To test whether some of the candidate proteins that we identified in our SILAC screen are directly targeted by Cdks, we generated recombinant bacterially expressed proteins for PHF6, FUBP1, and Smek2 and subjected them to *in vitro* kinase assays using recombinant Cdk2/cyclin A complexes. PHF6 and Smek2 were readily phosphorylated in these *in vitro* reactions (Fig 9F,G), while FUBP1 did not appear to be a Cdk substrate (Fig 9H). We identified potential conserved Cdk phosphorylation sites in both PHF6 (S154 and S155) and Smek2 (S840) that also were identified as phosphorylated residues in previous proteomic screens (Hornbeck *et al*, 2012). Mutation of these serine residues to alanine markedly reduced the Cdk-dependent phosphorylation of these proteins, suggesting that these residues are directly phosphorylated by Cdks (Fig 9F,G). We generated phospho-specific antibodies to monitor the phosphorylation of Smek2 S840 phosphorylation in cells. These antibodies were highly specific for S840 phosphorylation because they strongly cross-reacted with recombinant WT Smek2, but not with the S840A mutant Smek2 after incubation with ATP and Cdk2/cyclin A, as judged by immunoblotting (Fig 9I). We then used this antibody to test the Cdk-dependent phosphorylation of Smek2 S840 in DT40 cells. We found that inhibition of Cdk1 in Cdk2-knockout cells, but not cells expressing Cdk2, caused a rapid loss of signal in immunoblots probed with the phospho-specific S840 Smek2 antibody (Fig 9J). These data suggest that Smek2 is indeed a target of S-phase Cdk.

In summary, this proteomic screen in combination with ICP-based filtering provides a validated resource of a large variety of novel cell cycle-regulated chromatin-associated proteins. Our approach led us to discover a novel functional interplay between interphase Cdks and the chromatin association of a variety of novel candidate proteins such as Cdk1 itself, the APC/C regulatory subunit cdc20, the PP4 regulatory subunit Smek2, the helicase FUBP1, and the PHD domain-containing zinc finger protein PHF6. We show novel cell cycle phenotypes for the latter three candidates and find that at least two of these proteins are direct targets of interphase Cdks. We further used ICPs to highlight potential novel chromatin-associated cell cycle regulators among five of the least characterized proteins in the SILAC candidate list. This approach is validated by the discovery of novel cell cycle phenotypes following siRNA-mediated depletion of two out of these five candidates, namely TMA16 and OGFOD1. Further studies are necessary to elucidate the exact impact of this regulatory network on S-phase progression.

In a parallel study, we compared newly replicated chromatin with post-replicative chromatin using quantitative proteomics (Alabert *et al*, 2014). As expected, replication factors were strongly enriched in new chromatin. However, among the other candidates, we identified many non-canonical chromatin proteins, as was the case for our Cdk study. Again, ICPs provided an additional dimension that allowed us to focus on canonical chromatin proteins. When cloning three candidates with high ICPs and four with low ICPs, we found that all seven proteins behaved as expected and ultimately characterized a novel replication factor.

## Conclusion

Subcellular structures in general and not just interphase chromatin are highly interactive and dynamic and cannot fully be described as static, isolated features. Instead of providing a simple binary cut-off approach, we provide a means to filter datasets in a biologically meaningful manner that catches the dynamic nature of many processes. Our new concept has the potential to describe the plasticity of many biological structures by overcoming a number of limitations in current proteomic approaches. First, biological classifiers bypass the need for stringent biochemical purification and raise organelle definition from physical association to biological co-behavior. Next, the integration of many such classifiers into a probabilistic output reflects the dynamic composition of organelles. Probabilities could be valuable additions to public protein databases, essentially complementing manual, qualitative annotations with large-scale data-driven and quantitative information. In principle, these could be automatically updated from incoming large-scale experimental evidence. The resource of 7635 ICPs provided here can immediately focus candidate lists on archetypal chromatin functions and boost our understanding of chromatin-based processes as our biological validation demonstrates. We envisage that our approach can be applied to any organelle, compartment, or other complex biological structure.

## Materials and Methods

### Cell culture, SILAC labeling, and treatments

Human cell lines were grown in DMEM-based medium free of arginine and lysine (custom-made by AthenaES, Baltimore, MD, USA). The medium was supplemented with 10% dialyzed fetal bovine serum (Invitrogen), 0.2 mM arginine, and 0.79 mM lysine. For SILAC labeling (Ong *et al*, 2002) of human cells, medium was supplemented with unlabeled amino acids (light label), with arginine:HCl, 13C6 (Cambridge Isotope Laboratories), and lysine:HCl, 4,4,5,5-d4 (Sigma) for the medium label, or with arginine:HCl 13C6, 15N4 and lysine:HCl, 13C6, 15N2 (Sigma) for the heavy label. For biological classifiers, cells were treated as described in Table 1. HeLa, HEK293, and HepG2 cells were kind gifts from E. Schirmer, I. Stancheva, and A. Ladurner, respectively. HeLa Tet-On cells expressing a Flag-and HA-tagged version of macroH2A have been described (Timinszky *et al*, 2009). Cdk1as and cdk1as/cdk2^−/−^ DT40 cells were cultured for six cell cycles in SILAC RPMI 1640 medium (Thermo Fisher Scientific, Bremen, Germany) containing 10% dialyzed fetal bovine serum (F0392; Sigma), 2 mM L-glutamine, 0.1% β-mercaptoethanol, 100 U/ml penicillin, and 0.1 mg/ml streptomycin. In light conditions, the media were supplemented with 30 μg/ml l-arginine (Sigma) and 100 μg/ml l-lysine (Sigma) and in heavy conditions with 30 μg/ml l-arginine:HCl (U-13C6) and 100 μg/ml l-lysine:2HCl (4,4,5,5-D4) (Cambridge Isotope Laboratories). These cells were incubated at 39°C in a humidified cell culture chamber with 5% CO_2._ 10^8^ cells were treated with 10 μM of 1NMPP1 (gift from Dr. Hans Streicher, University of Sussex) for 2 h. All cell lines were tested for full label incorporation and lack of arginine to proline conversion. We routinely test our cell lines for mycoplasma contamination.

### Chromatin enrichment for proteomics (ChEP)

Typically, two 150-cm^2^ petri dishes of human cells were grown in SILAC light and heavy medium, respectively. 10^8^ DT40 cells were used per experimental condition. Cells were washed with PBS, then cross-linked *in vivo* with 1% formaldehyde in PBS for 10 min at 37°C, as for chromatin immunoprecipitation experiments (Solomon *et al*, 1988). Cross-linking was stopped by the addition of glycine to a final concentration of 0.25 M and incubation for 5 min at room temperature (RT). Cells were rinsed with PBS, scraped off, and harvested by centrifugation in 50 ml PBS (5 min, 423 *g*). The pellets were resuspended in 1 ml ice-cold cell lysis buffer (25 mM Tris, pH 7.4, 0.1% Triton X-100, 85 mM KCl; Roche protease inhibitors) and transferred to 2-ml test tubes. They were homogenized by carefully pipetting up and down with a 200-μl pipette tip. Lysed cells/nuclei were pelleted in a bench-top centrifuge at 2300 *g* for 5 min at 4°C. The supernatants (cytoplasm) were transferred to new tubes, and relative protein concentrations of light and heavy SILAC samples were estimated by Bradford assay. The nuclei pellets were resuspended in 500 μl cell lysis buffer containing 200 μg/ml RNase A and incubated for 15 min at 37°C. Equal amounts of nuclei from light and heavy SILAC samples were then pooled as estimated on the basis of cytoplasmic extract quantitation. Nuclei were collected by centrifugation at 2300 *g* for 10 min at 4°C. They were then resuspended in 500 μl of SDS buffer (50 mM Tris, pH 7.4, 10 mM EDTA, 4% SDS; Roche protease inhibitors) using hydrophobic pipette tips and incubated for 10 min at RT. Next, 1.5 ml of urea buffer (10 mM Tris, pH 7.4, 1 mM EDTA, 8 M urea) was added and mixed by inverting the tube multiple times. Centrifugation in a table-top centrifuge at full speed for 30 min (25°C) yielded a transparent, gel-like pellet. Using hydrophobic pipette tips, the pellet was resuspended again in 500 μl SDS buffer, mixed with 1.5 ml of urea buffer, and centrifuged for 25 min at full speed (25°C). The pellet was washed once more in 2 ml of SDS buffer. Finally, the pellet was covered with 0.5 ml of storage buffer (10 mM Tris, pH 7.4, 1 mM EDTA, 25 mM NaCl, 10% glycerol; Roche protease inhibitors) and sonicated in ice water to solubilize chromatin completely. After a final centrifugation step (30 min, full speed, 4°C), the amount of solubilized chromatin in the supernatant was quantified by Bradford assay. Formaldehyde cross-links were reversed by incubation with SDS–PAGE loading buffer for 30 min at 98°C.

### Preparation of other biochemical fractions

To prepare a nuclear lysate, cells were lysed using 0.1% Triton X-100, nuclei were isolated by centrifugation through a 30% sucrose cushion and homogenized using 1% SDS. A classic “chromatin pellet” used as biochemical classifier by comparing it with our improved method was prepared as described (Shiio *et al*, 2003).

### Mass spectrometry and data processing

Protein mixtures were digested in-gel with trypsin (Shevchenko *et al*, 2006), and peptides were fractionated by strong cation-exchange chromatography (2.1 × 200 mm polysulfoethyl A column; PolyLC, USA). Depending on the scale of the experiment, different gradients from buffer A (5 mM KH_2_PO_4_, 10% acetonitrile, pH 3.0) to 70% buffer B (1 M KCl in buffer A) were used. Peptide fractions were desalted on C18 StageTips (Rappsilber *et al*, 2003) and analyzed by LC-MS/MS on an LTQ-Orbitrap or LTQ-Orbitrap Velos (Thermo Fisher Scientific) as described (Ohta *et al*, 2010; Vagnarelli *et al*, 2011; Samejima *et al*, 2012). Mass spectra were processed using MaxQuant 1.3.0.5 (Cox & Mann, 2008; Cox *et al*, 2011) using default settings, except that ratio count was set to 1, minimum peptide length was set to 6, and only unique peptides were used for quantitation. Data were searched against the *Homo sapiens* complete proteome subset of the Uniprot database (UniProt Consortium, 2012), with canonical and isoform sequences downloaded on 13.07.2012. Chicken data were processed using the same MaxQuant conditions except considering unique and razor peptides for quantitation. They were searched against the *Gallus gallus* subset of Uniprot (complete proteome including unreviewed entries) downloaded on 25.09.2012. Statistically significant outliers were determined based on “Significance B” provided by the software Perseus 1.3.0.4 (http://www.perseus-framework.org), which takes protein intensities into consideration.

### Proteomics data deposition

The mass spectrometry proteomics data have been deposited to the ProteomeXchange Consortium (http://proteomecentral.proteomexchange.org) via the PRIDE partner repository (Vizcaíno *et al*, 2013) with the dataset identifiers PXD000492 (Cdk experiments) and PXD000493 (ICP classifier experiments).

### Multiclassifier chromatin proteomics (machine learning)

Log2 SILAC ratios and intensities of 7635 human proteins detected in some or all of our experiments (Supplementary Table 4) were combined using machine learning, essentially as described (Ohta *et al*, 2010). Here, the WEKA 3.6 implementation of RF (Breiman, 2001; Frank *et al*, 2004; Hall *et al*, 2009) was used via the KNIME 2.6.2 data analysis platform (Berthold *et al*, 2008). For this, the Weka data mining integration (version 2.6.1.0034734) was installed as a KNIME extension. First, a subset of 1068 proteins was manually defined as training proteins based on literature searches and Uniprot annotations. Training proteins fall in two classes, having either a reported function on chromatin (chromatin class) or a well-characterized function elsewhere in the cell and no indication for a chromatin-based activity (non-chromatin class). The RF algorithm is trained on these proteins and then ranks all 7635 proteins using a score from 0 to 1 depending on how similar they behave to the chromatin or the non-chromatin class. Optimal classification was achieved using RFs of 500 decision trees with unlimited tree depth and four random features. Under these conditions, 99.6% of training proteins were correctly classified (out-of-bag error 0.099). Training proteins were then cross-validated 100-fold using KNIME's X-partitioner node with stratified sampling. We manually annotated a further 4727 proteins into non-chromatin and chromatin classes, the latter including functional subcategories (“Lab internal category” in Supplementary Table 1). These proteins, together with the cross-validated 1068 training proteins, constitute our 5795 “evaluation proteins,” which we used to test RF performance based on ROC curves, yielding an area under the curve of 0.86 (please note that this is different from the ROC-like curves in Fig 2D, which show absolute protein numbers rather than true/false positive rates, thus illustrating that combining experiments also increases the number of proteins for which data are available). We provide our machine learning workflow as a supplementary KNIME archive file. Application of this workflow reproduces non-cross-validated RF scores (Supplementary Table 1) directly from Supplementary Table 4.

### Translation of RF scores into interphase chromatin probabilities (ICPs)

Integration of experiments by MCCP provides a machine learning score for all 7635 proteins (Supplementary Table 1), including the 5795 evaluation proteins and 1840 uncharacterized proteins. This score basically ranks the proteins according to how much their behavior resembles that of chromatin training proteins in the different experiments. The RF score has no meaning on its own, that is, outside the context of this study. To “translate” it into probabilities for chromatin function, we looked at the 5795 evaluation proteins that can be classified as chromatin or non-chromatin. We first calculated the percentage of chromatin proteins within nine overlapping ranges of RF score (0–0.2, 0.1–0.3, …) as shown by the bar chart in Fig 3A (the gray box corresponds to the RF score window 0.7–0.9). A sigmoid curve was then fitted through these percentage values, using SigmaPlot [Systat Software, USA) and the formula ICP = 1/(1 + exp(−a*RFscore + *b*)], where *a* and *b* are the parameters to be fitted. The fitted curve (adjusted *R*^2^ = 0.99, Fig 3A) was used to translate the RF score of all 7635 proteins in our dataset into an ICP. The number and size of RF score windows was shown to have very little impact on the final ICP values (Supplementary Fig S3). We therefore chose the simplest of the tested scenarios.

### Gene ontology analysis

Annotations for GO terms chromosome (GO:0005694) and cytoplasm (GO:0005737) were downloaded from the GO (Ashburner *et al*, 2000) database using QuickGO (Binns *et al*, 2009). Only qualifiers *contributes to*,*colocalizes with* and *none* were considered. For the analysis of uncharacterized proteins, the human GO associations file was downloaded from http://www.geneontology.org and processed using KNIME, considering the same qualifiers and only experimental evidence codes.

### Domain analysis

Information about protein domains was retrieved by querying Pfam (Punta *et al*, 2012) with the sequences of all 7635 proteins in this study. “Gathering threshold” was selected as cut-off; PfamB domains were not included.

### Identification of human–chicken orthologs

Orthologs were identified by querying ENSEMBL (Flicek *et al*, 2013) using the BioMart (Kasprzyk, 2011) data mining tool. We retrieved all chicken orthologs with homology type “one2one” from the Homo sapiens genes (GRCh37.p8) dataset of the Ensembl Genes 68 database. One-to-one orthologs that could not be unambiguously matched in this way were assigned manually based on sequence analysis. For this, the chicken protein sequence was compared to human protein sequence databases using the BLASTP tool (http://blast.ncbi.nlm.nih.gov). A human ortholog was assigned if the query sequence covered at least 50% of the human protein sequence and shared at least 50% identity.

### Protein interaction map

The interactome was built from human protein–protein interaction (PPI) databases. All reproducible Cdk-dependent outliers with an ICP above 0.1 were considered to build a PPI map using the software Cytoscape 2.8.3 (Smoot *et al*, 2011). Human PPIs from databases were retrieved using the plugin MiMI (Gao *et al*, 2009). Small-scale studies were considered and *in vivo* interactions or PPIs from direct complexes were displayed. The PPI map was complemented using data obtained from STRING (Franceschini *et al*, 2013) using experimental data and 0.4 confidence as parameters. Some PPIs were curated manually, and references are listed in Supplementary Table 5. Only outliers with protein interactions were shown in the protein interaction map and outliers with an ICP below 0.1 were added if they were shown to be a part of the indicated pathways. CDK substrates were downloaded from the database PhosphositePlus (Hornbeck *et al*, 2012; http://www.phosphosite.org).

### Immunofluorescence

To analyze Cdk regulation of FUBP1, PHF6, and Cdk1, asynchronous HeLa cells were treated for 2 h with 50 μM of roscovitine (Sigma). Cells were then treated with Triton X-100 according to the following conditions: PHF6, 0.1% Triton X-100 for 2 min; FUBP1, 0.1% for 1 min; Cdk1 0.2% for 1 min. Next, cells were fixed with 3.7% formaldehyde (Sigma) in PBS for 10 min. Cdc20-GFP HeLa BAC cells [gift from Tony Hyman (Poser *et al*, 2008)] and wild-type HeLa cells (to analyze Smek2) were synchronized in S-phase by adding 2 mM thymidine for 16–18 h, then released for 1 h before treatment with 50 μM roscovitine for 2 h. Next, S-phase cells were treated with 0.5% (Cdc20) or 0.2% (Smek2) Triton X-100 for 1 min, then fixed with 3.7% formaldehyde in PBS for 10 min. Cells were permeabilized in PBS–0.1% NP-40 for 10 min. Cells were then blocked in 3% BSA for 30 min and probed with primary antibodies for 1 h. Slides were rinsed in PBS and probed with Alexa Fluor secondary antibodies (Invitrogen) for 1 h. Slides were then rinsed in PBS, and coverslips were mounted using ProLong Gold mounting solution containing DAPI (Life Technologies). Smek2 and PHF6 rabbit polyclonal antibodies were purchased from Sigma, GFP rabbit polyclonal serum (to detect Cdc20-GFP) from Life Technologies, and FUBP1 rabbit polyclonal and fibrillarin and Cdk1 mouse monoclonal antibodies from Abcam.

Images were acquired on a microscope (DeltaVision) equipped with a UPLS Apochromat NA 1.40, 100× oil immersion objective (Olympus), standard filter sets (excitation 360/40, 490/20, and 555/28; emission 457/50, 528/38, and 617/40), and a camera (CoolSNAP HQ2; Photometrics). Images were obtained in softWoRx software (version 3.7.1) and exported as Photoshop (Adobe) files.

For quantitative data, DeltaVision files were imported into ImageJ. In the DAPI channel, the nucleus was highlighted by adjusting the threshold and the obtained regions of interest were reported to the FITC channel to measure the mean gray value. Data were then exported to Sigma Plot 12.0 (Systat Software), and after background subtraction, relative intensities were determined as a ratio relative to intensities measured in control cells and a box plot displayed the results.

### Flow cytometry

To deplete Smek2, PHF6, or FUBP1, HeLa cells were transfected with 10 nM of siRNA using Lipofectamine RNAimax (Invitrogen) for 72 h. RPE cells were transfected with siRNA pool (10 nM of each siRNA) targeted to OGFOD1, TMA16, C3ORF37, LYAR, and THYN1 for 72 h. Knockdown experiments were carried out with the following siRNA sequences:

5′-GCAUGAUAAAGCUCAAAUATT-3′ (s38848; Ambion. siPHF6_1) and

5′-CGCAUUUCUUGAGACUUAATT-3′ (SI00143248; Qiagen, siPHF6_2) for PHF6;

5′-GGAUUACAGGAGACCCAUATT-3′ (s16966; Ambion, siFUBP1_1) and

5′-CAUACAACCCUGCACCUUATT-3′ (SI04263147; Qiagen, siFUBP1_2) for FUBP1;

5′-CCAUCUAUAUUGCGUAGUATT-3′ (s32915, Ambion, siSmek2_1) and

5′-AGAAGCUCAGCAGAGUGAUTT-3′ (Qiagen, siSmek2_2) for Smek2;

On-targetplus SmartPool (L-021197-01; Thermo Scientific Dharmacon) for OGFOD1;

5′-GAGCAGAUUGAGUUACAUATT-3′ (s230546; Ambion),

5′-CCAUCCAUAUAGUAGAAAATT-3′ (s230547; Ambion) and

5′-GUGAACUAAUUGAAAGGUATT-3′ (s230545; Ambion) for TMA16;

5′-CUUCAUCUAUUUUCCUCAATT-3′ (s32441; Ambion),

5′-CUGUCGUAGUGAUACCGUATT-3′ (s32442; Ambion) and

5′-UCGACUUGGUGGUCAAAAATT-3′ (s32443; Ambion) for C3ORF37;

5′-GCAUAAGUGAAGAUCAGAATT-3′ (s31155; Ambion),

5′-CAUUGACUGCGGUAAAGAUTT-3′ (s31156; Ambion) and

5′-CAGUCAAUAAGGAACAGGATT-3′ (s31157; Ambion) for LYAR;

On-targetplus SmartPool (L-020704-02; Thermo Scientific Dharmacon) for THYN1.

AllStars Negative control from Qiagen (Cat.1027281) was used as a negative control. Smek2 polyclonal rabbit antibody (Abcam) was used to check siRNA efficiency by western blot, and FUBP1 and PHF6 antibodies were the same used in immunofluorescence experiments. α-Tubulin was used as loading control and detected with α-tubulin mouse monoclonal antibody from Abcam. To analyze replicating cells, 10 μM of EdU was added to the media for 1 h, and then the cells were collected and fixed with 70% ethanol. Cells were centrifuged at 380 *g* for 5 min, rinsed twice with PBS containing 3% BSA (BSA/PBS), and EdU-labeled DNA was stained according to the manufacturer's protocol (Click-iT EdU Alexa Fluor 647 Imaging kit; Invitrogen). The cells were then washed twice with PBS/BSA and resuspended in BSA/PBS solution containing 150 μg/ml RNase A and 5 μg/ml propidium iodide (PI). Cells were then analyzed for DNA content using a flow cytometer (FACSCanto; BD) and FACSDiva software (BD) to plot PI area versus cell counts and EdU staining intensity versus PI staining. The different cell cycle phases were gated using FACSDiva software, and measurements were exported to Microsoft Excel.

### Kinase assay

PHF6, FUBP1, and Smek2 cDNAs were obtained from mRNA isolated from HeLa cells by RT–PCR (SuperScript III One-Step RT-PCR system; Life Technologies) and cloned into a His-tag Gateway bacterial expression vector (a gift from Dr. Stephan Geley, University of Innsbruck, Austria). Cdk sites were modified using GeneArt site-directed mutagenesis kit (Life Technologies). Recombinant proteins were expressed in Rosetta bacteria, then purified with Ni-NTA beads (Qiagen) following the manufacturer's instructions. Next, purified recombinant proteins were incubated with Cdk2/cyclin A (a gift from Dr. Julian Gannon, CRUK Clare Hall, UK) for 20 min at 37°C in kinase buffer (50 mM MOPS pH 7.5, 5 mM MgCl_2_, 0.4 mM EDTA, 0.4 mM EGTA, 25 μM ATP, and 0.003 MBq γ-^32^P-ATP). The reaction was stopped by adding the SDS-PAGE loading buffer and was analyzed by western blot and autoradiography. Polyclonal rabbit phospho-S840 Smek2 antibody was generated by Eurogentec, Belgium.

## Supplementary information

for this article is available online: http://emboj.embopress.org
