# Supplementary material for: Proteomics of a fuzzy organelle: interphase chromatin
Source: EMBO J. 2014 Feb 17;33(6):648–64. doi: 10.1002/embj.201387614 (PMC3983682; doi:10.1002/embj.201387614)
Supplement: Supplementary file 1 [file embj0033-0648-sd1.pdf]

THE EMBO JOURNAL

Manuscript EMBO-2013-87614

Proteomics of a fuzzy organelle: interphase chromatin

Georg Kustatscher, Nadia Hegarat, Karen L.H. Wills, Cristina Furlan, Jimi-Carlo Bukowski-Wills, Helfried Hochegger and Juri Rappsilber

Corresponding author: Juri Rappsilber, University of Edinburgh

Review timeline:

Submission date:	06 December 2013
Editorial Decision:	23 December 2013
Revision received:	12 January 2014
Accepted:	14 January 2014

Editor: Hartmut Vodermaier

Please note that referee reports and author responses from previous review at another journal had been available in this case to the editors and referees at *The EMBO Journal*, and were taken into account in the decision process. Since the original reviews are not subject to EMBO's *Transparent Review Process* policy, only the reports and responses obtained directly at *The EMBO Journal* can be included in the transcript below.

Transaction Report:

(Note: With the exception of the correction of typographical or spelling errors that could be a source of ambiguity, letters and reports are not edited. The original formatting of letters and referee reports may not be reflected in this compilation.)

1st Editorial Decision 23 December 2013

Thank you again for transferring your manuscript together with the previous comments and responses to The EMBO Journal for our consideration. I have now heard back from two arbitrating referees, who had agreed to look both at the paper as well as at the previous referees' comments and your responses. I am pleased to inform you that both of them consider your methodology and dataset in principle of interest and publishable pending addressing of a limited number of specific concerns. In particular, it would appear important to satisfactorily address/clarify points 1 and 7 raised by our arbitrating referee 1.

As discussed previously, in addition to the changes requested by the arbitrating referees, please also incorporate into the final revised version the additional biological data you mentioned, and make sure to reorganize and reformat the manuscript for The EMBO Journal. This would entail, as also discussed before, having a proper introduction section (results and discussion can be combined, this is up to what you see best fit), inclusion of key Material & Methods in the main manuscript, and increasing the number of data and model figures/tables included in the main manuscript (including moving supplementary table 1 and supplementary figure 9 into the main part). We would also need you to reformat the reference list and in-text references according to journal style, and to include brief Author Contribution and Conflict of Interest statements (next to the Acknowledgment section). Finally, I would like ask you to provide (in your resubmission cover letter) 2-5 one-sentence 'bullet points' (complementary to the abstract) that summarize key aspects of the paper - they will

© European Molecular Biology Organization

accompany the online version of the article as part of a 'synopsis'. Please see the latest research articles on our renewed website (emboj.embopress.org) for examples - I am happy to offer further guidance on this if necessary.

Following these modifications, we should be happy to consider the study further for publication as a Resource Article in our journal. When uploading your final version (using the link provided below), please make sure to include a cover/response letter detailing the changes made to the manuscript and responding to each of the points of our arbitrating referees. Should you have any further questions in this regard, I'll be happy to answer/discuss them after the holiday break.

Thank you again for the opportunity to consider this study for publication in our journal! I look forward to receiving your revision.

REFEREE REPORTS

Referee #1:

Re manuscript entitled "Proteomics of a fuzzy organelle: interphase chromatin"

The manuscript by Kustatscher et al. describes an extension of the MCCP strategy, previously described by the same group in Cell in 2010. Whether the described improvements are significant or only marginal is more of an editorial decision. Having said that, the data are rather interesting, providing a very valuable resource for all people interested in interphase chromatin.
In addition to introducing the ICP score, Kustatscher et al. perform several follow up experiments on four selected proteins in order to confirm their findings (in addition, they provide an accepted manuscript in which their novel ICP approach was already utilized).
The manuscript is potentially of interest for the EMBO J readership; however the following issues have to be addressed before acceptance can be considered.

Major issues:
1) The section describing Figure 1d is misleading. The authors state that "indeed, even a combination of 6 such 'biochemical classifiers' is outperformed (Fig. 1d)." Given the fact that they compared 6 different biochemical classifiers (representing ~9 different stages) against many more biological classifiers (representing ~20 different stages, many of which with repeated data analysis, i.e. better data quality), I would expect that the biological classifiers outperform the biochemical classifiers - however, this is an unfair comparison and does not say anything about the superiority of the biological vs. biochemical classifiers. Considering that more than 2 times as many stages were used for the biologically-motivated classification, the performance increase is actually quite marginal and raises the question whether the additional performance increase justifies the additional labor. It might also be that I misunderstood this section and that the authors tried to compare apples with apples, but either way this paragraph should be clarified or significantly revised.

2) Page 6/22, top: it is not clear where suddenly the 7653 proteins are coming from. Until then, the authors discussed 5795 proteins. Please clarify this issue. In addition, it has to be clarified how the lists of 5795 and 7653 proteins are related. Is one the subset of the other?

3) The point of Fig. 3d is not completely clear. A low ICP can indicate general non-chromatin association as well as temporal non-chromatin association, i.e. it is a hotchpotch of proteins. Instead it might be better to look at the ICP scores of proteins that are known to be chromatin-associated only during mitosis and compare the ICP score distribution with the one from bona fide non-chromatin-associated proteins.

4) It is not clear how those 4 proteins for further testing were selected. Based on the ICP scores of the selected proteins, I would have expected some non-chromatin-associated proteins and it would have been interesting to include some negative control experiments, in order to emphasize the relevance of the follow-up experiments in Fig. 4d to 4h.

5) The authors keep referring to ROC curves. However, Fig. 1d is not a ROC curve. It is a quasi-ROC curve or a ROC-like curve; this has to be corrected in the text.

6) Figure 4d and 4e: the special role of PHF6 vs the other three test proteins should be properly discussed in the manuscript. For instance, why did they use different quantitative measure in Figure 4e for PHF6 vs. Smek2 etc.

7) Material and Methods/MCCP section: it seems that the authors keep integrating the training data into the test data, i.e. there is no clear separation between these two datasets. For proper validation, they have to be separated. Combining the test and training data might also be the reason for this excessively correct classification of 99.6 % which seems to be 'too good to be true' and might be an indication of overfitting. Thus, I strongly suggest repeating the RF analysis with clearly separated training and test datasets. In addition, it is not clear how the first set of 1068 proteins were selected for the manually defined training proteins - this also has to be clarified.

Minor issues:

1) The term 'fuzzy organelle' should be clearly defined.

2) An abbreviated version of Suppl. Table 1 should be in the main text.

3) Since there are many CDKs, the authors should clearly indicate when they are referring to cdk1 (see e.g. middle of page 9/22: "We also included Cdk that has a low...".

Referee #2:

After reading the full paper and reviewers´ concerns, I feel that I do not have sufficient expertise to critically evaluate the soundness of the machine learning method used in this manuscript.

The technical aspect of the proteomics part in the paper appears solid. However, as two of the reviewers pointed, there is poor description of the datasets. Instead of providing a proper description of the data, the authors´ response is "For this study it was important that we cover a wide range of conditions, but we did not describe each of these experiments in detail" and " We have indeed not provided a legend to match experimental conditions in Table S1....."
This is important to address this point (i.e. providing proper details of experimental conditions for the data reported in the supplemental tables) if the manuscript is published as a resource paper.

1st Revision - authors' response 12 January 2014

Referee #1:

The manuscript by Kustatscher et al. describes an extension of the MCCP strategy, previously described by the same group in Cell in 2010. Whether the described improvements are significant or only marginal is more of an editorial decision. Having said that, the data are rather interesting, providing a very valuable resource for all people interested in interphase chromatin.
In addition to introducing the ICP score, Kustatscher et al. perform several follow up experiments on four selected proteins in order to confirm their findings (in addition, they provide an accepted manuscript in which their novel ICP approach was already utilized).
The manuscript is potentially of interest for the EMBO J readership; however the following issues have to be addressed before acceptance can be considered.

Major issues:
1) The section describing Figure 1d is misleading. The authors state that "indeed, even a combination of 6 such 'biochemical classifiers' is outperformed (Fig. 1d)." Given the fact that they compared 6 different biochemical classifiers (representing ~9 different stages) against many more biological classifiers (representing ~20 different
stages, many of which with repeated data analysis, i.e. better data quality), I would expect that the biological classifiers outperform the biochemical classifiers - however, this is an unfair comparison and does not say anything about the superiority of the biological vs. biochemical classifiers. Considering that more than 2 times as many stages were used for the biologically-motivated classification, the performance increase is actually quite marginal and raises the question whether the additional performance increase justifies the additional labor.
It might also be that I misunderstood this section and that the authors tried to compare apples

with apples, but either way this paragraph should be clarified or significantly revised.

We agree that our comparison between biological and biochemical classifiers in Figure 1d (now Figure 2D) is misleading and have removed the corresponding sentence from the text. Please note that the performance increase seen in Figure 2D is not the only difference. Figure S1 shows that biological classifiers correct artefacts of biochemical experiments. This links to the fact that biological classifiers do not depend on the quality of the isolation procedure. That opens up proteomics analyses for such cellular structures that cannot be enriched in a sufficiently specific way.

2) Page 6/22, top: it is not clear where suddenly the 7653 proteins are coming from. Until then, the authors discussed 5795 proteins. Please clarify this issue. In addition, it has to be clarified how the lists of 5795 and 7653 proteins are related. Is one the subset of the other?

We observed 7635 proteins of which 5795 are characterised and 1840 are uncharacterised. We further clarified this in the text and in the legend of figure 3.

3) The point of Fig. 3d is not completely clear. A low ICP can indicate general non-chromatin association as well as temporal non-chromatin association, i.e. it is a hotchpotch of proteins. Instead it might be better to look at the ICP scores of proteins that are known to be chromatin-associated only during mitosis and compare the ICP score distribution with the one from bona fide non-chromatin-associated proteins.

To increase clarity we have made Fig. 3d into its own Figure now, accompanied with a paragraph in the text that has its own subheading.

4) It is not clear how those 4 proteins for further testing were selected. Based on the ICP scores of the selected proteins, I would have expected some non-chromatin-associated proteins and it would have been interesting to include some negative control experiments, in order to emphasize the relevance of the follow-up experiments in Fig. 4d to 4h.

We chose the 5 initial candidates in the validation of the Cdk SILAC results based on their ICPs and SILAC ratios as mentioned on page 10. We then validated the ICP method with a set of uncharacterised proteins with high ICP values. We also have cloned seven uncharacterised proteins in the accompanying manuscript by Alabert et al. and show that all of the three positive and four negative predictions were correct.

5) The authors keep referring to ROC curves. However, Fig. 1d is not a ROC curve. It is a quasi-ROC curve or a ROC-like curve; this has to be corrected in the text.

This has been corrected.

6) Figure 4d and 4e: the special role of PHF6 vs the other three test proteins should be properly discussed in the manuscript. For instance, why did they use different quantitative measure in Figure 4e for PHF6 vs. Smek2 etc.

We have discussed this on page 10 as follows: "In the case of PHF6 we observed a sequestration of the protein in the nucleus, and a release on the non-nucleolar chromatin after Cdk inhibition. In all other cases we observed an simple increase or decrease of chromatin association following Roscovitine treatment that correspondent with the respective SILAC ratio in the proteomic data set."

7) Material and Methods/MCCP section: it seems that the authors keep integrating the training data into the test data, i.e. there is no clear separation between these two datasets. For proper validation, they have to be separated. Combining the test and training data might also be the reason for this excessively correct classification of 99.6 % which seems to be 'too good to be true' and might be an indication of overfitting. Thus, I strongly suggest repeating the RF analysis with clearly separated training and test datasets.

We have used 101 random forests in our analysis. One random forest was trained based on the full set of training proteins and used to classify all of the non-training proteins. This random forest yielded "correct classification of 99.6 %" for the training, showing that the random forest was sufficiently large to learn the training data. For the remaining 100 random forests we used each time 99% of the training data for training and classified the remaining 1%. This is referred to as 100-fold cross-validation. For all the training proteins we used data from the particular of the 100 random forests in which they were not used as training proteins, i.e. part of the 1% training proteins that were left out. In summary, training proteins double in their role, serving to train 100 random forests and to test one random forest in which they were not used as a training protein. The 100-fold cross-validation ensures that we did not over train.

In addition, it is not clear how the first set of 1068 proteins were selected for the manually defined training proteins - this also has to be clarified.

1068 proteins were manually defined as training proteins based on literature searches and Uniprot annotations. This is described in the materials and methods section.

Minor issues:
1) The term 'fuzzy organelle' should be clearly defined.
This has been added to the introduction.

2) An abbreviated version of Suppl. Table 1 should be in the main text.
We have added this as new Table 1.

3) Since there are many CDKs, the authors should clearly indicate when they are referring to cdk1 (see e.g. middle of page 9/22: "We also included Cdk that has a low...".
We have clarified this point with the following definition on page 9: “Below, we will indicate the combination of Cdk1 and Cdk2 as “Cdk”.” And have referred to Cdk1 and Cdk2 specifically when these proteins were specifically involved (as in the example pointed out by the reviewer on page 10).

Referee #2:

After reading the full paper and reviewers´ concerns, I feel that I do not have sufficient expertise to critically evaluate the soundness of the machine learning method used in this manuscript.

The technical aspect of the proteomics part in the paper appears solid. However, as two of the reviewers pointed, there is poor description of the datasets. Instead of providing a proper description of the data, the authors´ response is "For this study it was important that we cover a wide range of conditions, but we did not describe each of these experiments in detail" and " We have indeed not provided a legend to match experimental conditions in Table S1....."
This is important to address this point (i.e. providing proper details of experimental conditions for the data reported in the supplemental tables) if the manuscript is published as a resource paper.

The first resource we provide is the protocol to conduct chromatin enrichment for proteomics (ChEP), which is described in full detail.
We furthermore see our ICP values for 7635 proteins to be a resource, which is provided in Supplementary Table S1. We provide the full experimental detail needed to reproduce our meta-analysis leading to these ICP values. This includes all MS raw data, SILAC ratios and intensities and the machine learning workflow.
The treatments used for individual SILAC experiments are summarized in Table 1. We have not provided a legend to match this table with the specific data columns in Supplementary table S4. The present manuscript can be fully understood and reproduced without such a legend, as we do not make use of individual outlier proteins found in any of these SILAC experiments. However, these experiments represent a significant proportion of biological projects in our lab. We would therefore like to publish these specific data in separate studies on the biological roles of these chromatin modulations.

Finally, we provide as a third resource a list of CDK-induced protein association changes to chromatin. We give a thorough description of all experimental details.

© European Molecular Biology Organization
