# Supplementary material for: Proteomics of a fuzzy organelle: interphase chromatin
Source: EMBO J. 2014 Feb 17;33(6):648–64. doi: 10.1002/embj.201387614 (PMC3983682; doi:10.1002/embj.201387614)
Supplement: Supplementary file 2 [file embj0033-0648-sd2.pdf]

SUPPLEMENTARY FIGURE S1

A

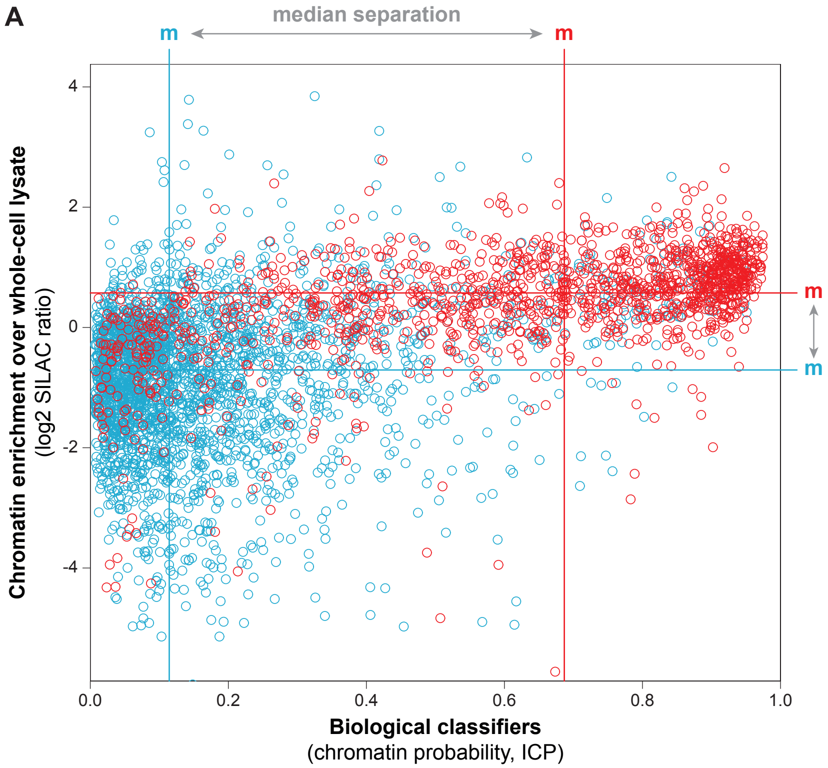

B

Uniprot ID	Biological classifiers (ICP)	Chromatin enrichment (log2 SILAC)	Protein Name
P69905	0.33	3.85	Hemoglobin alpha chain
Q9Y2S2	0.14	3.79	Gulonate 3-dehydrogenase
Q14703	0.14	3.38	Endopeptidase S1P
Q93084-2	0.16	3.27	SR calcium ATPase 3
Q9NTJ4	0.42	3.27	Alpha-mannosidase 2C1
O75054	0.09	3.25	Ig superfamily member 3
Q9Y2H0	0.20	2.88	Disks large-associated protein 4
O43295	0.63	2.83	Rho GTPase-activating protein 14
O15438-4	0.42	2.80	Multi-specific organic anion transporter D
Q5K4L6	0.10	2.75	Fatty acid transport protein 3

Supplementary Figure S1. Biological classifiers reveal artefacts of biochemical enrichment. a, A combination of biological classifiers provides a better bulk separation of chromatin (red) and non-chromatin proteins (blue) than biochemical enrichment of chromatin over a whole-cell lysate. Lines correspond to population medians. Biological classifiers (see Table 1 for full list) were integrated using machine learning and transformed into interphase chromatin probabilities (ICPs), in an analogous procedure to the one described in the main text for all classifiers (biological and biochemical). The scatterplot shows 1398 chromatin and 2636 non-chromatin proteins. **b,** The 10 most highly enriched contaminants in biochemically purified chromatin. Based on biological behavior, 9 of these are more likely to be non-chromatin than chromatin (chromatin probability < 0.5).
