# Supplementary material for: Proteomics of a fuzzy organelle: interphase chromatin
Source: EMBO J. 2014 Feb 17;33(6):648–64. doi: 10.1002/embj.201387614 (PMC3983682; doi:10.1002/embj.201387614)
Supplement: Supplementary file 3 [file embj0033-0648-sd3.pdf]

SUPPLEMENTARY FIGURE S2

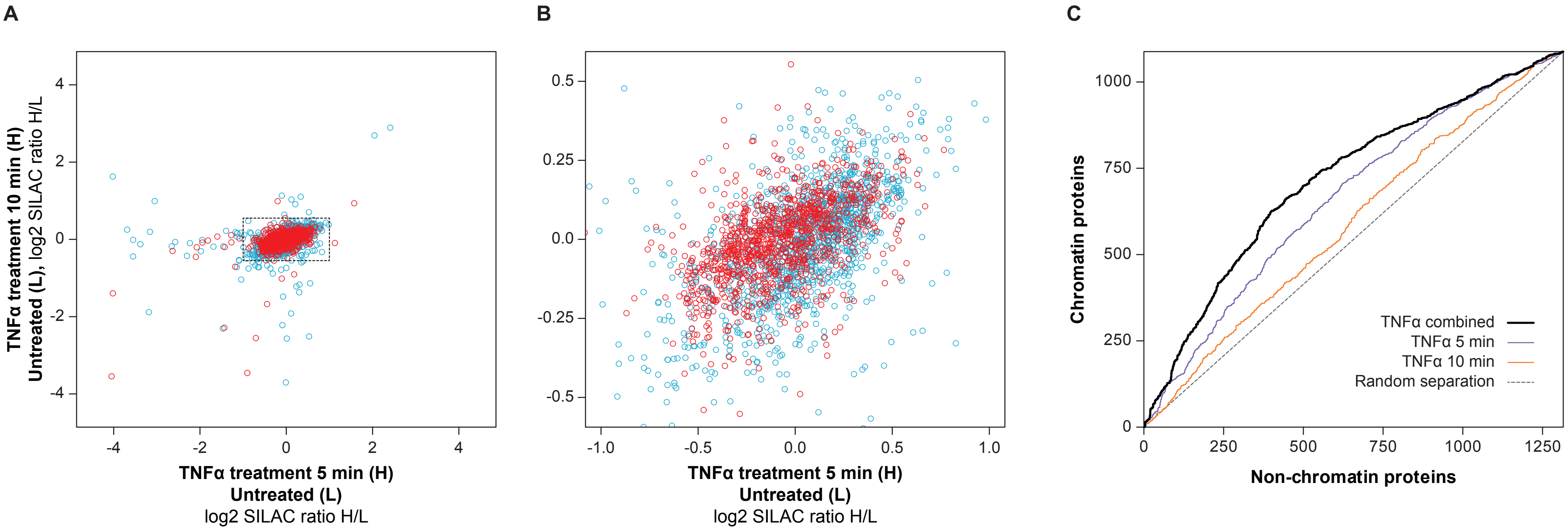

Supplementary Figure S2. Similar physiological alterations provide complementary information about interphase chromatin. a, Chromatin fractions of cells treated with TNFα for 5 min and 10 min, analysed by quantitative proteomics (heavy SILAC label: H; light SILAC label: L). The bulk of proteins appears to be unaffected by either condition. **b,** Magnification of the region outlined in panel a reveals that chromatin (red) and non-chromatin proteins (blue) overall react distinctly to TNFα treatment, as evident from the small bulk offset between the two groups. **c,** Machine-learning is capable of extracting this minute amount of information from both individual experiments as shown by ROC curves. Integration of the two experiments greatly enhances performance, even though these physiological conditions are very similar. The plots show 1088 known chromatin and 1315 non-chromatin proteins.
