# Supplementary material for: Proteomics of a fuzzy organelle: interphase chromatin
Source: EMBO J. 2014 Feb 17;33(6):648–64. doi: 10.1002/embj.201387614 (PMC3983682; doi:10.1002/embj.201387614)
Supplement: Supplementary file 4 [file embj0033-0648-sd4.pdf]

SUPPLEMENTARY FIGURE S3

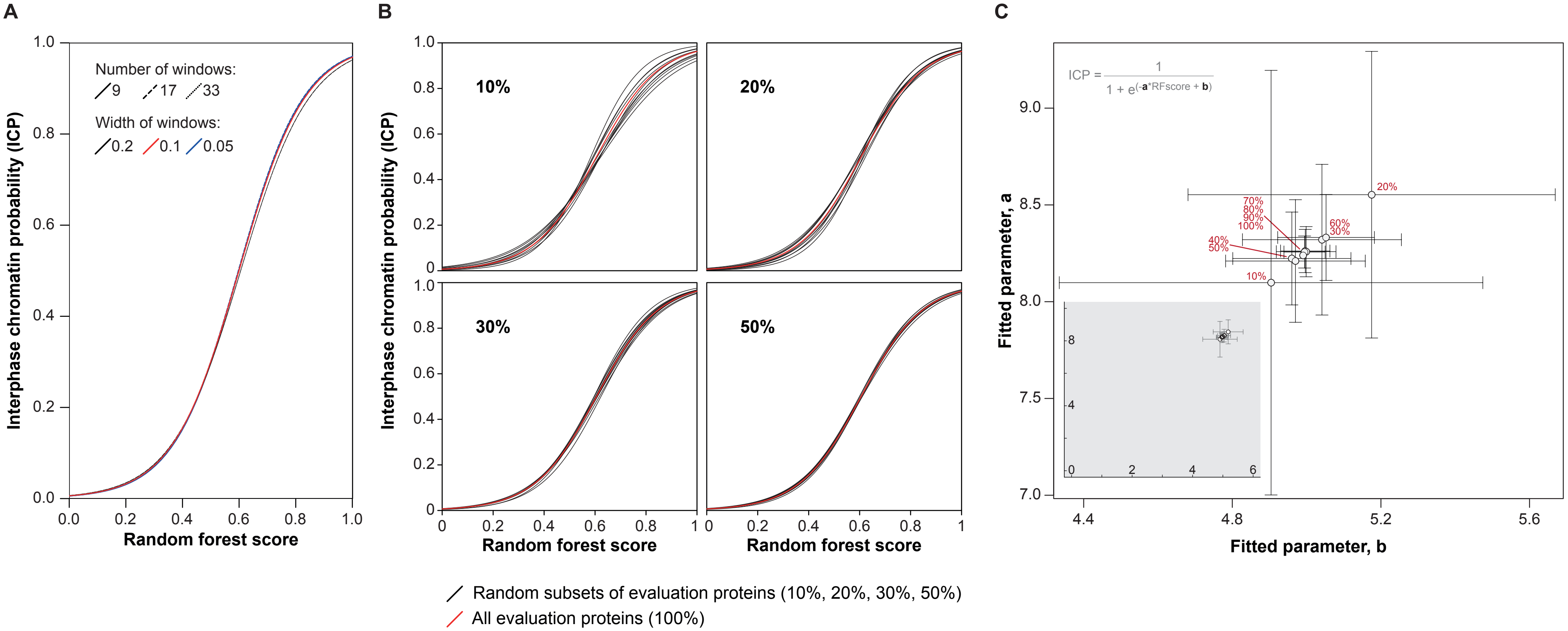

Supplementary Figure S3. Translation into interphase chromatin probabilities (ICPs) is robust and reproducible. a, Calculating ICPs from random forest scores is nearly unaffected by the number and size of windows used. We therefore chose the simplest scenario of 9 windows and width 0.2 (shown in Figure 3A). **b,** The choice of evaluation proteins does not have a major impact on ICP calculation. Randomly selected subsets of 10% (similar to 10-fold cross-validation), 20%, 30% or 50% of our 5795 evaluation proteins result in very similar translation curves, keeping n = 10 for consistency. The random subsets (black curves) were tested and compared to the curve derived using all evaluation proteins (red). Note that already with 30% (1738 randomly selected evaluation proteins) all 10 samples result in nearly identical curves, i.e. the actual choice of evaluation proteins becomes irrelevant. Random subsets were drawn using the row sampling node in KNIME. **c,** The fitted curve parameters from (b) and additional random subsets are displayed. For each percentage, the average fitted parameters of 10 different random selections are shown, error bars represent the standard deviation over these 10 different samples. The inset displays the full scale of the plot, demonstrating that the parameters quickly converge.
