# Supplementary material for: Proteomics of a fuzzy organelle: interphase chromatin
Source: EMBO J. 2014 Feb 17;33(6):648–64. doi: 10.1002/embj.201387614 (PMC3983682; doi:10.1002/embj.201387614)
Supplement: Supplementary file 5 [file embj0033-0648-sd5.pdf]

SUPPLEMENTARY FIGURE S4

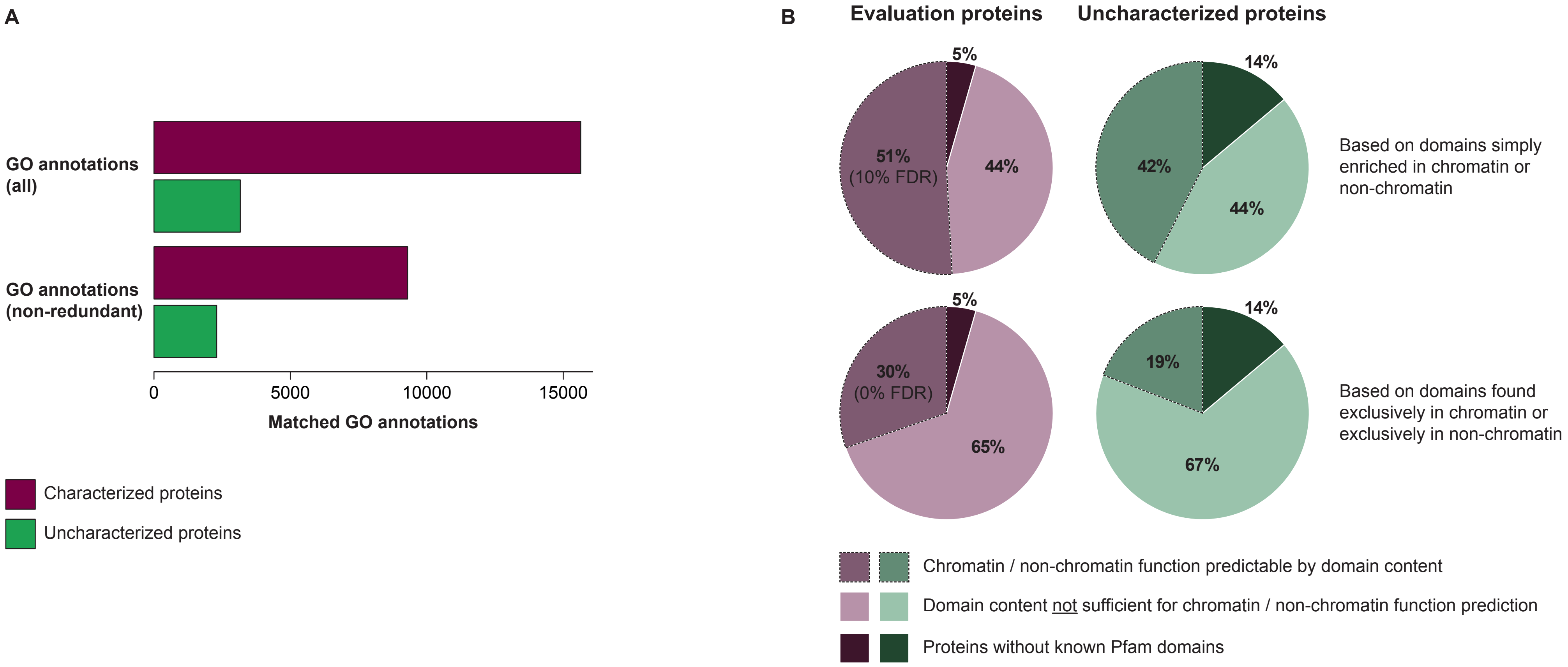

Supplementary Figure S4. Proteins annotated as uncharacterized in this study have low GO coverage and weak domain-based prediction. We assign ICPs to 1840 proteins which were manually classified as *uncharacterized* based on Uniprot and literature searches. **a,** This classification is supported by the low number of Gene Ontology (GO) annotations available for these proteins compared to an equivalent random subset of our evalutation proteins. Only annotations with experimental evidence were considered. **b,** Protein domains can only partially predict functional categories for uncharacterized proteins and indeed also for characterized proteins. Domains were assigned a “chromatin” or “non-chromatin” label according to how often they occur in our 1823 chromatin or 3972 non-chromatin evaluation proteins. Only domains that occur in more than 5 evaluation proteins and that are either enriched (upper part) or exclusive (lower part) to either category were used for protein function prediction. Considering domains that are enriched in chromatin or non-chromatin allows for the prediction of 51% of our evaluation proteins (10% false discovery rate, FDR). Domains found exclusively in chromatin or exclusively in non-chromatin proteins, which consequently predict all of them correctly (0% FDR), occur in only 30% of evaluation proteins. Of the 1840 uncharacterised proteins described in this study, only 42% (with a 10% FDR) or 19% (with a 0% FDR) could be placed into a chromatin or non-chromatin category based on the domains they contain. Note that in contrast to ICPs these would be simple binary categories.
