# Supplementary material for: Proteomics of a fuzzy organelle: interphase chromatin
Source: EMBO J. 2014 Feb 17;33(6):648–64. doi: 10.1002/embj.201387614 (PMC3983682; doi:10.1002/embj.201387614)
Supplement: Supplementary file 6 [file embj0033-0648-sd6.pdf]

SUPPLEMENTARY FIGURE S5

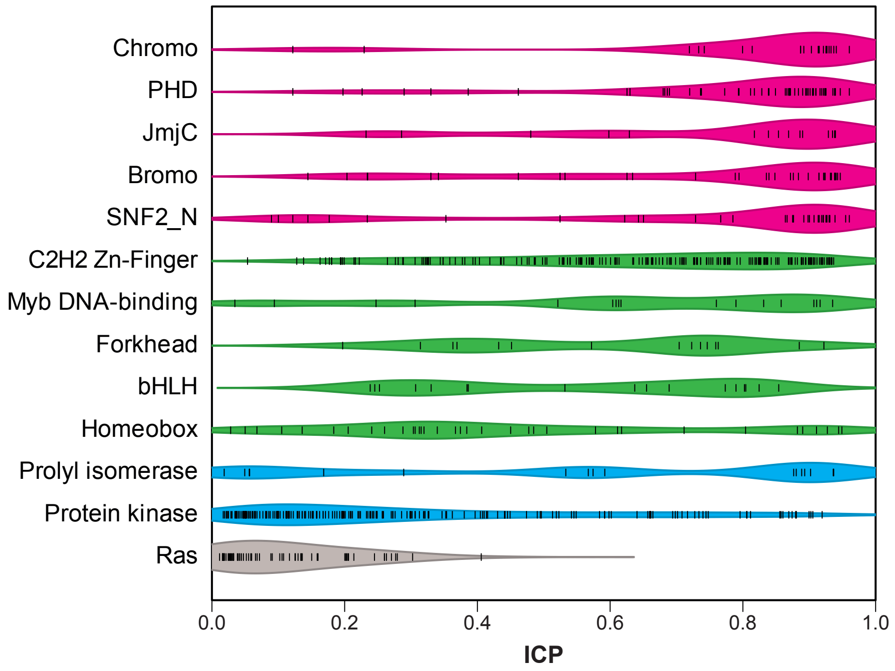

Supplementary Figure S5. Interphase chromatin probabilities (ICPs) and protein domains. Proteins with core chromatin domains (magenta) invariably have high ICPs, whereas proteins with sequence-specific DNA binding domains (green) are dispersed in agreement with their optional, regulated chromatin functions. ICP suggests enzymes (blue) that may act on chromatin. Ras domain (grey) is unlikely to be involved with chromatin. Note that high ICPs may suggest regulators of chromatin function among divergent enzyme families, including kinases, and indicate candidates for human homologues of histone prolyl isomerases.
