# Supplementary material for: Proteomics of a fuzzy organelle: interphase chromatin
Source: EMBO J. 2014 Feb 17;33(6):648–64. doi: 10.1002/embj.201387614 (PMC3983682; doi:10.1002/embj.201387614)
Supplement: Supplementary file 7 [file embj0033-0648-sd7.pdf]

SUPPLEMENTARY FIGURE S6

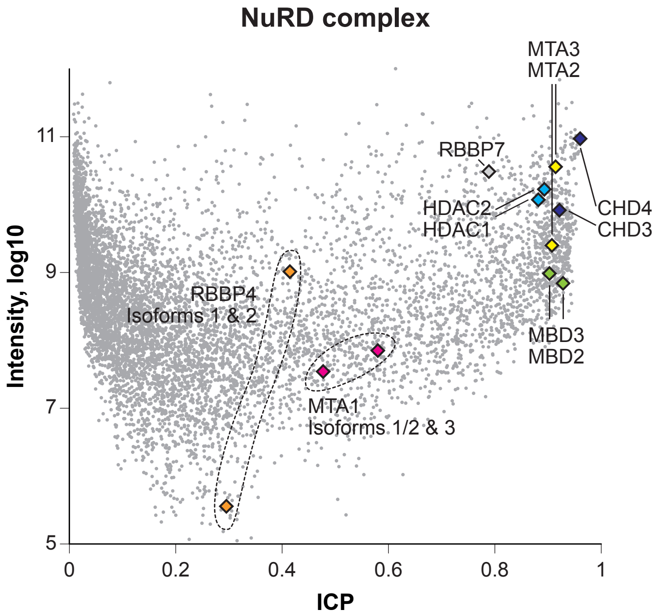

Supplementary Figure S6. ICP describes multifunctional subunits of the NuRD complex. NuRD subunits are multifunctional proteins, which is reflected in considerable divergence in ICP within the complex. However, splice forms and paralogous genes of individual NuRD subunits appear to have related functions as judged by their similar ICP. Most subunits of the nucleosome remodelling and histone deacetylase NuRD complex have very high chromatin ICPs, with the exception of RBBP4 and MTA1. This is consistent with some MTA1 isoforms having cytoplasmic activities (Kumar *et al.*, *Nature* **418**, 654-657 (2002)), in contrast to MTA2 and MTA3. Note that the detected isoforms of both RBBP4 and MTA1 were independently assigned similar ICPs. A clustering of functionally related proteins is apparent also among NuRD subunits with high ICPs.
