# Supplementary material for: Proteomics of a fuzzy organelle: interphase chromatin
Source: EMBO J. 2014 Feb 17;33(6):648–64. doi: 10.1002/embj.201387614 (PMC3983682; doi:10.1002/embj.201387614)
Supplement: Supplementary file 13 [file embj0033-0648-sd13.docx]

Table S1: Interphase chromatin probabilities for 7635 human proteins

Table S2: SILAC ratios and statistical analysis of Cdk-dependent chromatin composition

Table S3: Significant Cdk outliers with human one-to-one orthologs and ICPs

Table S4: Input file for the random forest analysis

Table S5: Literature and database references for protein interaction networks
