# Supplementary material for: Proteomics of a fuzzy organelle: interphase chromatin
Source: EMBO J. 2014 Feb 17;33(6):648–64. doi: 10.1002/embj.201387614 (PMC3983682; doi:10.1002/embj.201387614)
Supplement: Supplementary file 14 [file embj0033-0648-sd14.pdf]

SUPPLEMENTARY FIGURE S7

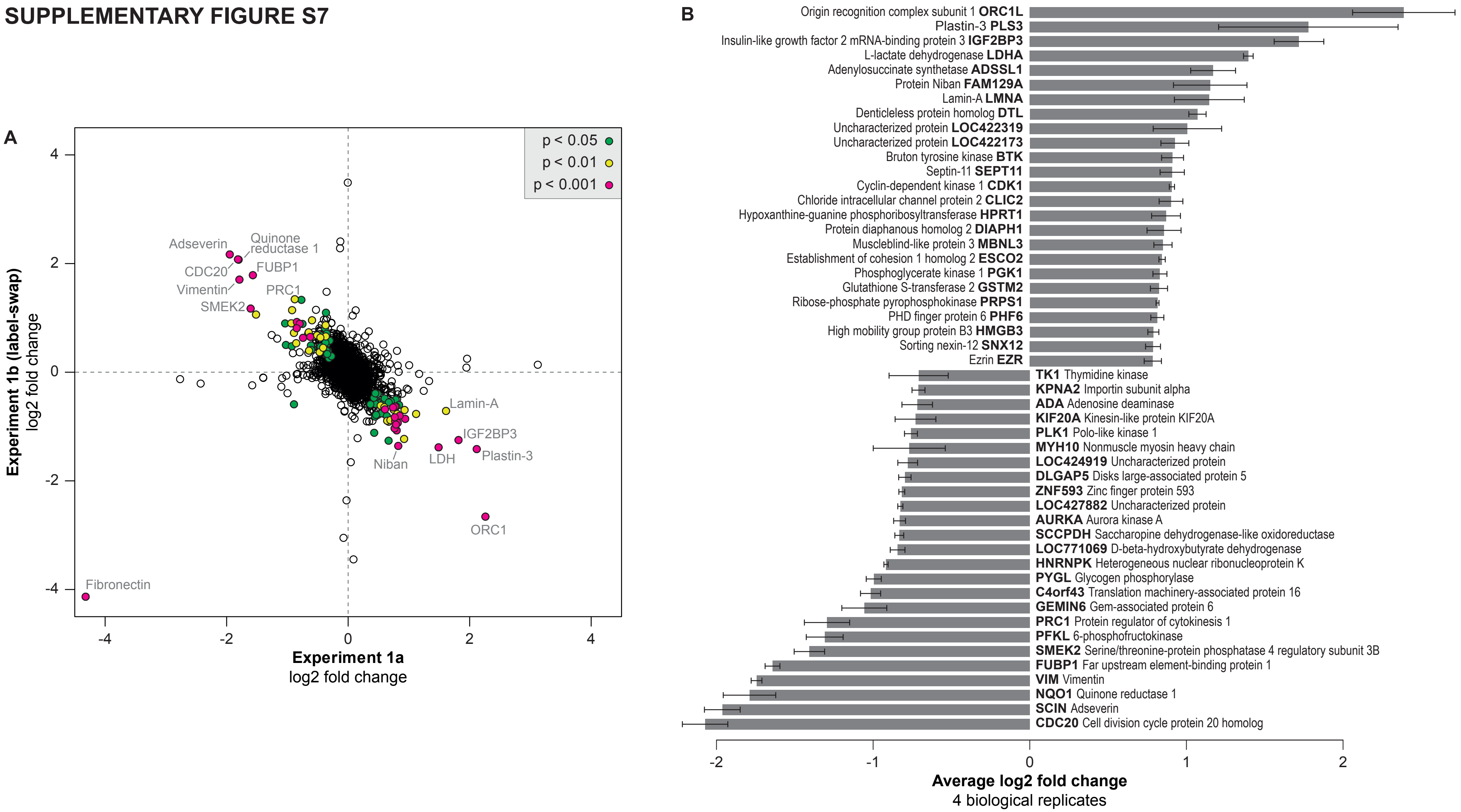

Supplementary Figure S7. A quantitative proteomics screen for Cdk regulation of interphase chromatin. a, SILAC ratios of 2046 proteins quantified in two of the total four biological replicate experiments. Filled circles represent statistically significant outliers, including the positive control Orc1. The label-swap control identifies fibronectin as unlabelled external contaminant. **b,** The 25 most highly up- or downregulated proteins across the four experiments. Error bars show the standard error of the mean. All proteins are significantly changed ($p < 0.05$) in at least three of four replicates.
